# EphA2–YES1–ANXA2 pathway promotes gastric cancer progression and metastasis

**DOI:** 10.1038/s41388-021-01786-6

**Published:** 2021-05-03

**Authors:** Linfeng Mao, Weijie Yuan, Kaimei Cai, Chen Lai, Changhao Huang, Yi Xu, Shangwei Zhong, Chen Yang, Ran Wang, Pengwei Zeng, Heyuan Huang, Zhikang Chen, Zihua Chen

**Affiliations:** 1 Department of General Surgery, Xiangya Hospital, Central South University (CSU), Hunan Changsha, China; 2 Department of Gastrointestinal Surgery, Xiangya Hospital, CSU, Hunan Changsha, China; 3 The Hunan Provincial Key Lab of Precision Diagnosis and Treatment for Gastrointestinal Tumor, Xiangya Hospital, CSU, Hunan Changsha, China; 4 Department of Colorectal and Anus Surgery, Xiangya Hospital, CSU, Hunan Changsha, China; 5 International Joint Research Center of Minimally Invasive Endoscopic Technology Equipment & Standardization, Xiangya Hospital, CSU, Hunan Changsha, China

**Keywords:** Gastric cancer, Metastasis

## Abstract

Erythropoietin-producing hepatocellular receptor A2 (EphA2) is a key member of the receptor tyrosine kinase (RTK) family, while YES Proto-Oncogene 1 (YES1) is a non-receptor tyrosine kinase (nRTK) and annexin A2 (ANXA2) belongs to the calcium-dependent phospholipid-binding protein family annexins. Here, we show that EphA2, YES1, and ANXA2 form a signal axis, in which YES1 activated by EphA2 phosphorylates ANXA2 at Tyr24 site, leading to ANXA2 activation and increased ANXA2 nuclear distribution in gastric cancer (GC) cells. Overexpression (OE) of YES1 increases, while knockdown (KD) of YES1 or ANXA2 decreases GC cell invasion and migration in vitro and tumor growth in mouse models. Reexpression of wildtype (WT) rather than mutant ANXA2 (Tyr24F) in ANXA2 knockdown (ANXA2-KD) GC cells restores YES1-induced cell invasion and migration, while neither WT nor mutant ANXA2 (Tyr24F) can restore cell invasion and migration in YES1-KD GC cells. In addition, the activation of EphA2–YES1–ANXA2 pathway is correlated with poor prognosis. Thus, our results establish EphA2–YES1–ANXA2 axis as a novel pathway that drives GC invasion and metastasis, targeting this pathway would be an efficient way for the treatment of GC.

## Introduction

Gastric cancer (GC) is a common malignancy of the digestive tract. There are more than one million new cases of GC every year worldwide, ranking the sixth in new cases of cancer and the second in the cancer caused death [1, 2]. Currently, the early diagnosis rate of GC is still very low, many patients are already in the advanced stage at the time of diagnosis [3, 4]. Traditional precision surgery and postoperative chemotherapy do not significantly decrease the recurrence and improve the 5-year-survival rate of advanced GC patients [5, 6]. Understanding the mechanisms that underlie the pathogenesis and progression of GC is therefore needed to develop novel therapeutic approaches.

EphA2 is a key member of the receptor tyrosine kinase (RTK) family that plays important roles in multiple physiological and pathological processes. Accumulating evidence suggests that EphA2 is overexpressed in various cancers, including colorectal cancer [7], breast cancers [8], GC [9], and nasopharyngeal carcinoma [10]. As a RTK on the cell membrane, EphA2 can regulate many oncological behaviors of cancer cells through different signaling pathways [11–13]. We had reported that EphA2 promotes the progress of GC through the Wnt/β-catenin pathway [14, 15].

YES1 is a member of SRC kinase family (SRCs) of nRTKs that is widely existing in the cytoplasm. The SRCs share a common modular structure, all of which contains the kinase domain. The kinase domain is flanked by two regulatory regions [16], YES1 is catalytically activated when its Tyr426 is phosphorylated, while YES1 is enzymatically inhibited when its Tyr537 is phosphorylated [16, 17]. Accumulating evidences suggest that YES1 plays important roles in growth, invasion, and metastasis of various tumors [18–21]. However, the roles of YES1 and the underlying mechanisms in GC development are unclear.

ANXA2 is a member of the calcium-mediated phospholipid-binding protein family annexins, which comprise a highly conserved C-terminal domain and a variable N terminal domain that is unique to each annexin [22]. The amino-terminal domain of ANXA2 contains three important phosphorylation sites, which are crucial to its subcellular locations and biological function. Ser phosphorylation of ANXA2 seems to be an essential event of the secretory process [23], while Tyr24 phosphorylation of ANXA2 (p-ANXA2-Y24) has been related to the localization of ANXA2 to the endocytic membrane system and the nucleus, and is associated with malignant transformation, EMT, tumor invasion, and metastasis [23–26]. ANXA2 has recently been linked to the metastasis of several types of cancer, such as prostate [27], colorectal [28], and breast cancer [29]. High expression of ANXA2 promotes the development of GC and is negatively correlated with the clinical prognosis [30, 31].

Here, we show that EphA2, YES1, and ANXA2 form a signal axis, in which YES1 activated by EphA2 phosphorylates ANXA2 at Tyr24 site, resulting in ANXA2 activation and increased ANXA2 nuclear distribution in GC cells, which may drive GC invasion and metastasis.

## Result

### EphA2 interacts with YES1 and phosphorylates YES1 at Tyr426 site

**Fig. 1:**
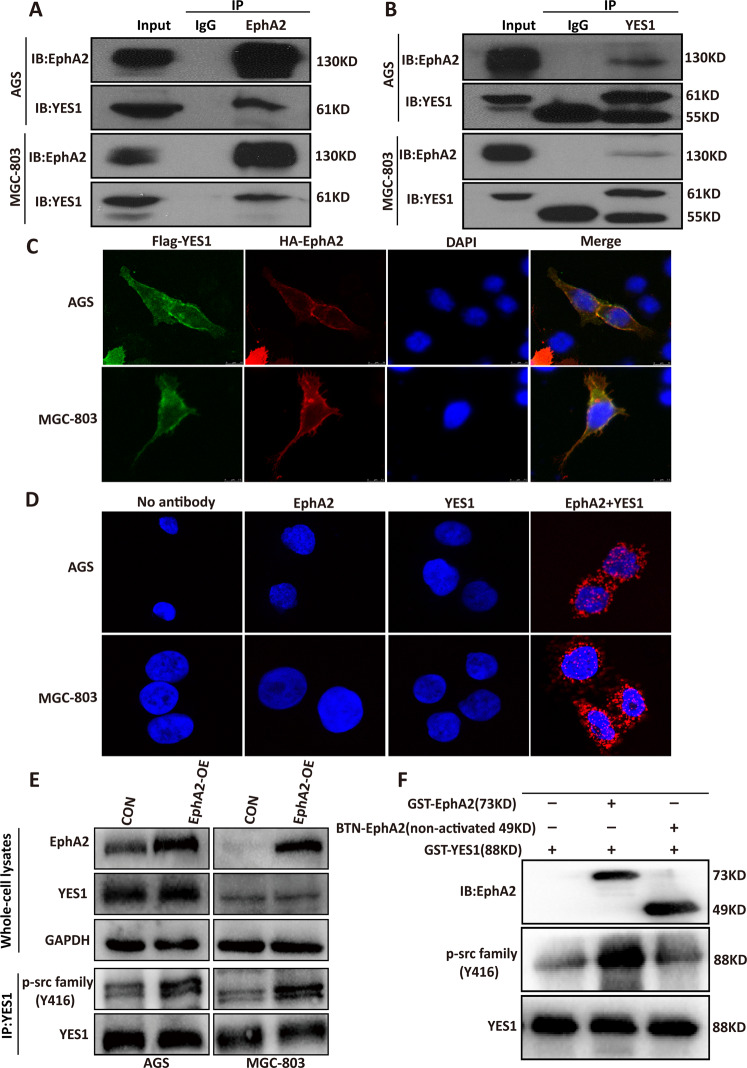
EphA2 interacts with YES1 and phosphorylates YES1 at Tyr426 site. **A**, **B** EphA2 co-immunoprecipitation with YES1 (**A**) or YES1 co-immunoprecipitation with EphA2 (**B**) in indicated GC cells. **C** Immunofluorescent staining of HA-EphA2 and Flag-YES1 in indicated GC cells. **D** Proximity ligation assay (PLA) for the interaction of EphA2 and YES1 in indicated GC cells. **E** Western blot analysis for the expression of indicated proteins in the whole cell lysates or the expression of p-SRC family Y416 in immunoprecipitated YES1 protein in control and EphA2 overexpression (EphA2-OE) AGS and MGC-803 cells. **F** Recombinant human GST-YES1 was incubated with recombinant human active GST-EphA2 or nonactivated BTN-EphA2 at 30 °C for 30 min. The reaction mixtures were analyzed by immunoblot with anti-p-SRC family Y416, EphA2, and YES1 antibodies.

We had demonstrated that EphA2 plays important roles in GC growth and progression [9, 14], however, the underlying mechanisms are unclear. To identify the interacting proteins of EphA2, we transfected AGS cells with control or plasmid expressing HA-EphA2, 48 h later cells were collected and immunoprecipitation (IP) was performed using anti-HA antibody. The HA-EphA2-IP protein complex was subjected to proteomic analysis. We found that YES1 is one of the major proteins identified in the HA-EphA2-IP protein complex (Table S1). Co-immunoprecipitation (Co-IP) experiments confirmed the interaction between endogenous EphA2 and YES1 (Fig. 1A, B), as well as the interaction between exogenous HA-EphA2 and Flag-YES1 in MGC-803 and AGS cells (Fig S1A, B). It should be noted that Lyn, as one of SRCs, is also in the list of the HA-EphA2-IP proteins, but its specific peptides and score used to predict the possibility of its interaction with EphA2 are very low. The interaction of EphA2 with Lyn (Fig. S2A) and other SRCs, such as Src (Fig. S2B), Fyn (Fig. S2C), and Lck (Fig. S2D) could not be confirmed by Co-IP assay. Confocal immuno-fluorescence microscopy imaging analysis showed the colocalization of exogenous HA-EphA2 and Flag-YES1 in MGC-803 and AGS cells (Fig. 1C). Consistently, proximity ligation assay (PLA) further supported the interaction between endogenous EphA2 and YES1 (Fig. 1D).

YES1 is a member of SRCs of nRTKs that is involved in various physiological and pathological processes. Phosphorylation of SRCs at Tyr416 (corresponding to Tyr426 of YES1) in the activation loop of the kinase domain upregulates enzyme activity [32]. To investigate whether EphA2 phosphorylates and activates YES1, AGS and MGC-803 cells were transfected with HA-EphA2 expression plasmid, 48 h later cells were collected and subjected to IP with YES1 antibody to pull down the endogenous YES1 protein, followed by immunoblot using Phospho-SRC family Tyr416 antibody. We found that the Tyr426 phosphorylation of endogenous YES1 increased in EphA2 overexpression (OE) cells compared with control cells (Fig. 1E). Furthermore, in vitro kinase assay showed that active GST-EphA2 but not the nonactive BTN-EphA2 could phosphorylate YES1 at Tyr426 site (Fig. 1F). Altogether, these results suggest that EphA2 interacts with YES1 and phosphorylates YES1 at Tyr426 site.

### YES1 promotes GC cell proliferation and migration

**Fig. 2:**
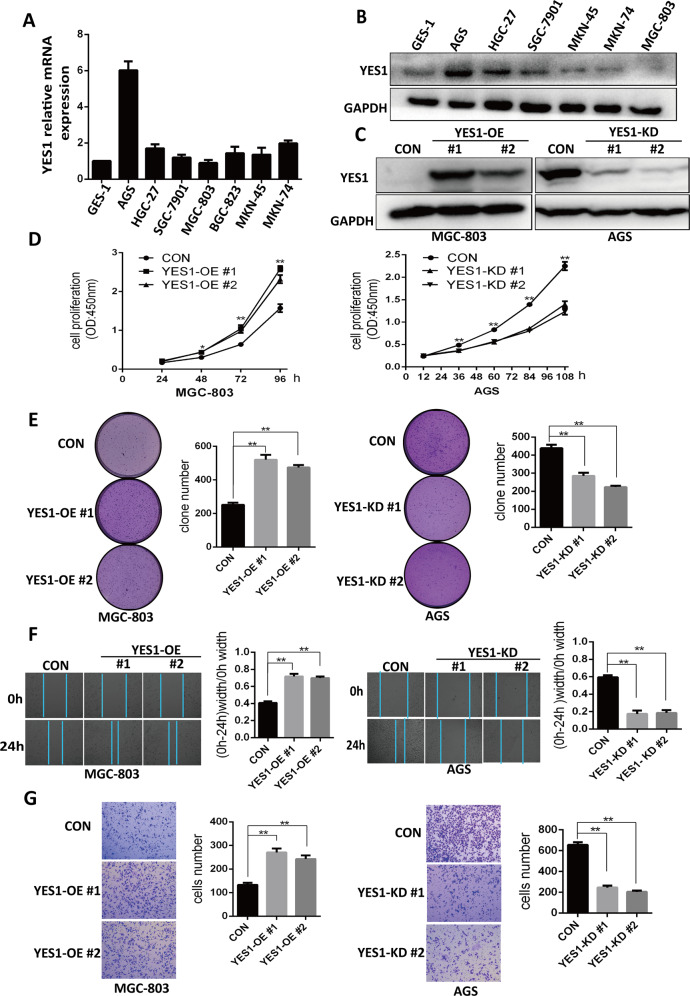
YES1 promotes GC cell proliferation, migration, and invasion. **A** qRT-PCR analysis for YES1 mRNA expression in GES-1 and different GC cell lines. **B** Western blot for the expression of indicated proteins in GES-1 and different GC cell lines. **C** Western blot for the expression of indicated proteins in control and YES1-OE MGC-803 cells or YES1-KD AGS cells. **D** CCK8 analysis for the proliferation of control and YES1-OE MGC-803 cells (left panel), or control and YES1-KD AGS cells (right panel). **E** Representative images and the quantification data of soft agar colony formation assay for control and YES1-OE MGC-803 cells (left panel), or control and YES1-KD AGS cells (right panel). **F** Representative images and the quantification data of scratch wound-healing assay for control and YES1-OE MGC-803 cells (left panel), or control and YES1-KD AGS cells (right panel) at 0 and 24 h after wound scratch. **G** Representative images and the quantification data of trans-well invasion assay for control and YES1-OE MGC-803 cells (left panel), or control and YES1-KD AGS cells (right panel).

To investigate the role of YES1 in GC, we examined YES1 mRNA and protein expression in a group of human GC cell lines and normal gastric epithelium (GES-1) cell line using qRT-PCR and western blot (WB). We found that the expression of YES1 was relatively high in AGS cells and low in MGC-803 cells (Fig. 2A, B). Based on the YES1 expression level, AGS cells was chosen for establishment of YES1 stable knockdown (KD) cells while MGC-803 cells for YES1 stable OE cells. The expression of YES1 in YES1 knockdown (YES1-KD) or YES1 overexpression (YES1-OE) clones was confirmed by WB (Fig. 2C). We compared the cell proliferation rates, colony formation, migration and invasion among YES1-OE, YES1-KD, and their control cells using CCK8 assay, soft agar colony formation assay, scratch wound-healing assay, and trans-well invasion assay. We found that YES1 OE significantly increased MGC-803 cell proliferation (Fig. 2D, left panel), colony formation (Fig. 2E, left panel), migration, and invasion ability (Fig. 2F, G, left panel). while YES1 KD significantly decreased AGS cell proliferation (Fig. 2D, right panel), colony formation (Fig. 2E, right panel), migration, and invasion (Fig. 2F, G, right panel). These results suggest that YES1 plays important roles in GC cell proliferation, migration, and invasion.

### YES1 promotes GC growth and metastasis in mouse models

**Fig. 3:**
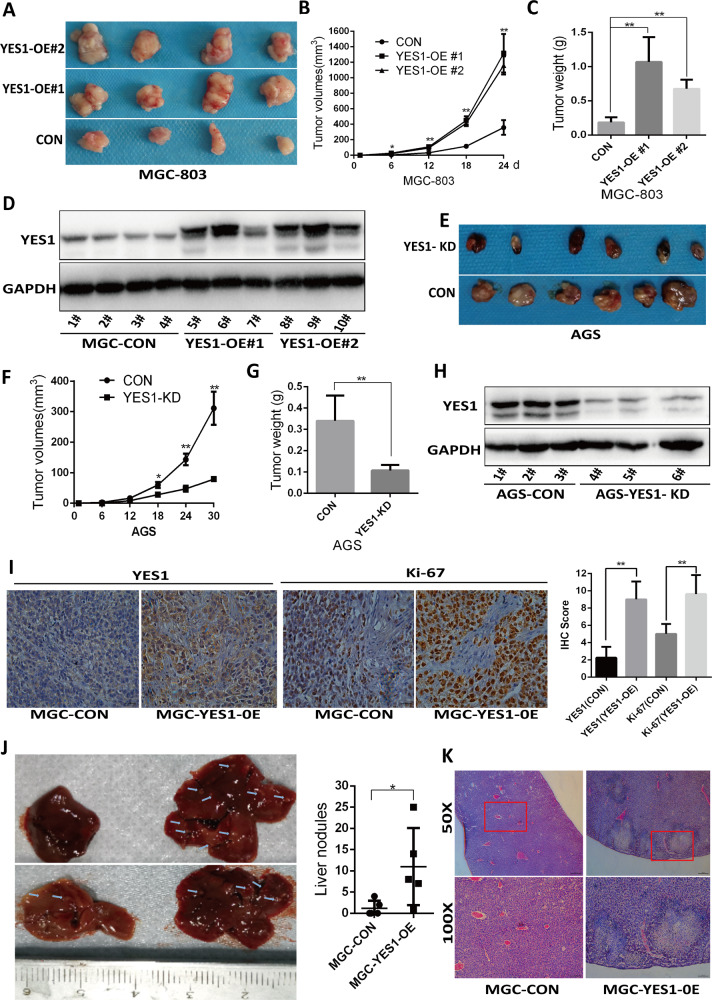
YES1 promotes GC xenograft tumor development in mouse models. **A**–**D** YES1-OE and control MGC-803 cells were inoculated subcutaneously in NOD-SCID mice, tumor images are shown (**A**), tumor size was measured every 6 days (**B**). Twenty-four days later mice were sacrificed and tumor were collected and weighted (**C**), and the expression of indicated protein in tumors was analyzed by western blot (**D**). **E**–**H** YES1-KD and control AGS cells were inoculated subcutaneously in NOD-SCID mice, tumor images are shown (**E**), and tumor size was measured every 6 days (**F**). Thirty days later mice were sacrificed and tumor were collected and weighted (**G**), and the expression of indicated protein in tumors was analyzed by western blot (**H**). **I** Immunohistochemical staining of YES1 and Ki-67 in indicated mouse xenograft tumors. **J**, **K** YES1-OE and control MGC-803 cells were injected into the spleen of nude mouse, thirty days later mice were sacrificed, and metastatic nodules in the liver were counted (**J**). Representative images of metastatic foci in paraffin-embedded liver sections stained by hematoxylin and eosin. Arrows indicate the liver metastatic nodules (**K**).

To further examine the role of YES1 in GC development, YES1-OE and control MGC-803 cells or YES1-KD and control AGS cells were inoculated into NOD-SCID mice and tumor development was monitored. Twenty-four or thirty days later mice were sacrificed and tumor were collected, weighted. We found that OE of YES1 significantly increased GC xenograft tumor development (Fig. 3A–D), while KD of YES1 significantly decreased GC xenograft tumor development (Fig. 3E–H). The OE of YES1 in YES1-OE xenograft tumors (Fig. 3D) and the KD of YES1 in YES1-KD xenograft tumors (Fig. 3H) were confirmed by WB. Furthermore, the expression of proliferation marker Ki-67 examined by immunohistochemistry (IHC) was significantly increased in YES1-OE xenograft tumors (Fig. 3I).

To examine the role of YES1 in metastasis, a liver metastasis mouse model was employed. YES1-OE and control MGC-803 cells were injected into the spleen of nude mouse, 30 days later, mice were sacrificed, and tumor metastatic nodules in the liver were counted. We found that mice inoculated with YES1-OE MGC-803 cells had more metastasis nodules in the liver than that inoculated with control MGC-803 cells (Fig. 3J, K). These results further support that YES1 drives GC growth and metastasis.

### YES1 phosphorylates ANXA2 at Tyr24 site that leads to ANXA2 activation and increased ANXA2 nuclear distribution

**Fig. 4:**
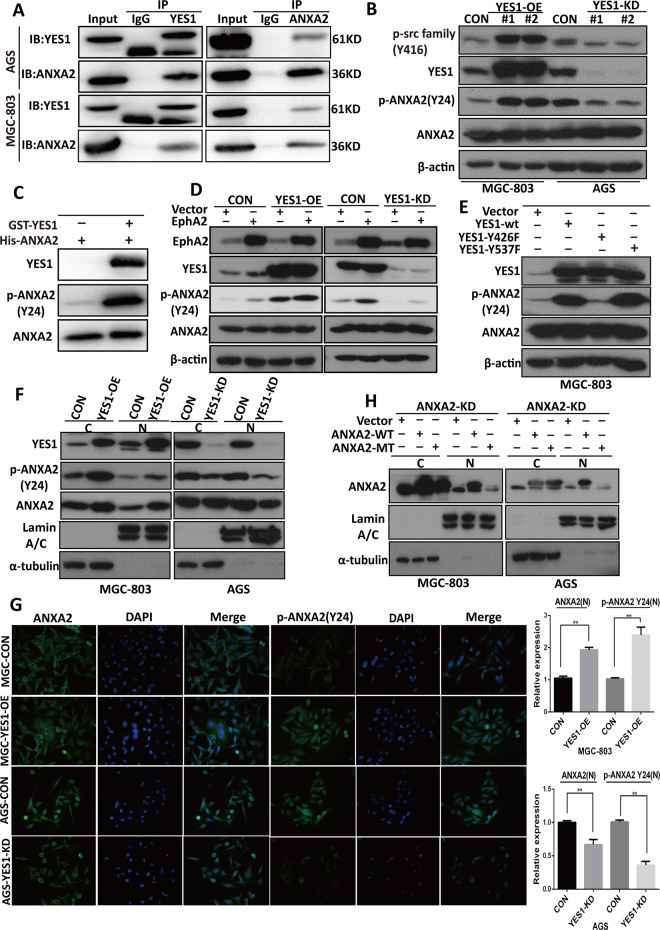
The phosphorylation of ANXA2 at Tyr24 by YES1 increases ANXA2 nuclear distribution. **A** YES1 co-immunoprecipitation with ANXA2 or ANXA2 co-immunoprecipitation with YES1 in indicated GC cells. **B** Western blot for the expression of indicated proteins in control and YES1-OE MGC-803 cells, or control and YES1-KD AGS cells. **C** Recombinant human His-ANXA2 was incubated with the absence or presence of recombinant human active GST-YES1 at 30 °C for 30 min. The reaction mixtures were analyzed by immunoblotting with anti-p-ANXA2 (Tyr24), ANXA2, and YES1 antibodies. **D** Western blot for the expression of indicated proteins in control, YES1-OE and YES1-KD cells transiently transfected with control or EphA2 overexpression plasmid for 48 h. **E** Western blot for the expression of indicated proteins in MGC-803 cells transiently transfected with control or WT-YES1, YES1-Y426F, and YES1-Y537F expression plasmid for 48 h. **F** Western blot for the expression of indicated proteins in cytoplasmic and nuclear extracts of control and YES1-OE MGC-803 cells or control and YES2-KD AGS cells. **G** Immunofluorescent staining of indicated proteins in control and YES1-OE MGC-803 cells or control and YES1-KD AGS cells. **H** Western blot for the expression of indicated proteins in cytoplasmic and nuclear extracts of ANXA2-KD cells transiently transinfected with control, ANXA2-WT or MT-ANXA2-Y24F expression plasmid for 48 h.

To search the phosphorylated substrates of YES1, we performed global phosphorproteomic and compared the phosphoproteins in YES-OE, YES-KD, and their control GC cells. We found that the Tyr24 phosphorylation level of ANXA2 was significantly upregulated in YES-OE cells while was downregulated in YES-KD cells (Table S2), however, OE or KD of YES1 had no effect on the expression of total ANXA2 protein (Table S2-1). Co-IP experiments confirmed the interaction between endogenous YES1 and ANXA2 (Fig. 4A), as well as the interaction between exogenous Flag-YES1 and HA-ANXA2 in MGC-803 and AGS cells (Fig. S3A, B). WB analysis further confirmed that the expression of p-ANXA2-Y24 was significantly increased in YES1-OE cells while significantly decreased in YES1-KD cells, although OE or KD of YES1 had no effect on the expression of total ANXA2 protein (Fig. 4B). In vitro kinase assay showed that active GST-YES1 could phosphorylate ANXA2 at Tyr24 site (Fig. 4C). As YAP1 is one major target of YES1, to investigate whether YES1-induced Tyr24 phosphorylation of ANXA2 is mediated by YAP1, YAP1-KD, and control AGS cells were transfected with YES1 plasmid and the expression of p-ANXA2-Y24 was examined. We found that YES1 increased the expression of p-ANXA2-Y24 in both control and YAP1-KD AGS cells, suggesting that YES1-induced p-ANXA2-Y24 is no dependent on YAP (Fig. S4A). These results suggest that YES1 interacts with ANXA2 and phosphorylates ANXA2 at Tyr24 site.

To investigate whether EphA2 induces p-ANXA2-Y24 and whether YES1 mediates EphA2-induced ANXA2 phosphorylation, YES1-OE, YES1-KD and their control GC cells were transfected with EphA2 expression or control plasmids, 48 h later cells were collected for WB analysis. We found that EphA2 OE significantly increased the expression of p-ANXA2-Y24 in control cells, further increased in YES1-OE cells, while had minor effect on YES1-KD cells (Fig. 4D). Furthermore, MGC-803 cells were transfected with plasmid expressing wildtype (WT), kinase dead (Y426F) or constitutively active (Y537F) YES1 proteins and the expression of p-ANXA2-Y24 was examined. We found that WT-YES1 and YES1-Y537F significantly increased the expression of p-ANXA2-Y24, while YES1-Y426F had no such effect (Fig. 4E).

Considering that phosphorylation modification of ANXA2 affects its function and cellular distribution [23, 33], we investigated the influence of the YES1-induced Tyr24 phosphorylation status on ANXA2 cellular distribution. Cell fractionation experiment showed that as compared with controls both nuclear ANXA2 and nuclear p-ANXA2-Y24 protein were much higher in YES1-OE cells while much lower in YES1-KD cells (Fig. 4F). Immunofluorescent staining of ANXA2 and p-ANXA2-Y24 also showed that the expression of nuclear ANXA2 and nuclear p-ANXA2-Y24 protein was increased in YES1-OE cells while decreased in YES1-KD cells (Fig. 4G). To examine whether the p-ANXA2-Y24 is essential for its nuclear distribution, we established ANXA2-KD AGS and MGC-803 cells by infecting with control or lentivirus engineered to express ANXA2 shRNA which specifically targets ANXA2 3′-UTR and selected by puromycin. ANXA2-KD cells were then transfected with control, wildtype (WT-ANXA2) or mutant-type ANXA2 (MT-ANXA2-Y24F) plasmids and the cytoplasmic and nuclear protein were extracted for WB analysis. We found that the exogenous WT-ANXA2 protein was expressed in both cytoplasm and nucleus while the MT-ANXA2-Y24F protein was mainly located in cytoplasm (Fig. 4H), suggesting that the Tyr24 phosphorylation of ANXA2 can increase its nuclear distribution. Together, these results suggest that YES1 interacts with ANXA2 and phosphorylates ANXA2 at Tyr24 site, leading to ANXA2 activation and increased ANXA2 nuclear distribution.

### ANXA2 mediates YES1-induced GC cell invasion and migration, but not YES1-induced GC cell proliferation

**Fig. 5:**
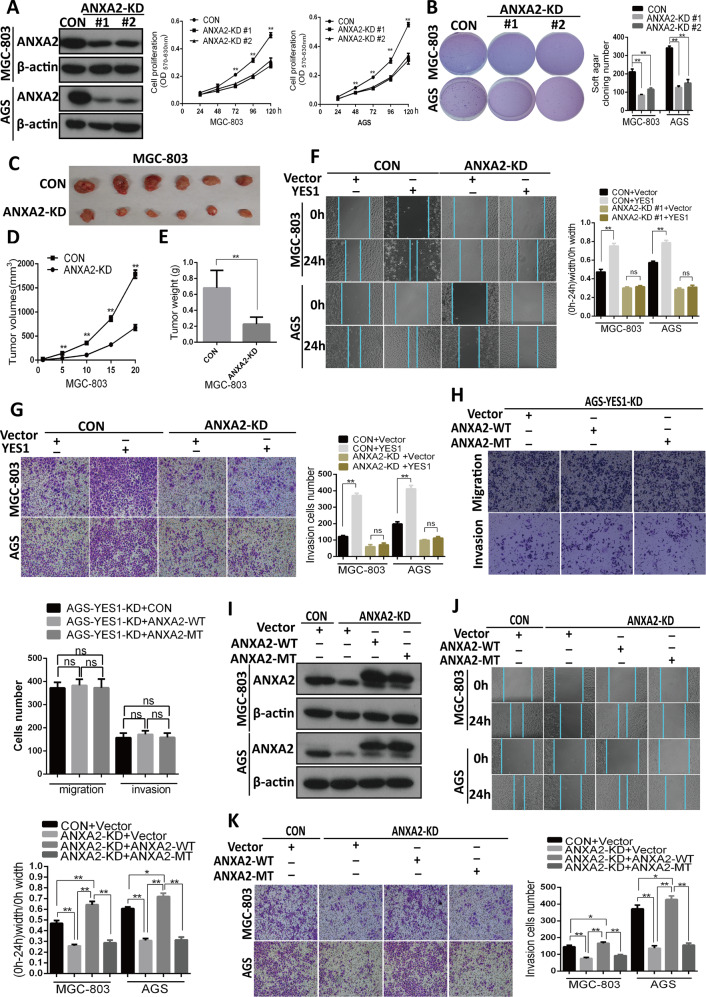
ANXA2-Tyr24 phosphorylation by YES1 promotes GC cell migration and invasion. **A** Western blot for the expression of indicated proteins in ANXA2-KD AGS and MGC-803 cells. MTT analysis for cell proliferation rates of control and ANXA2-KD MGC-803 and AGS cells. **B** Representative images of soft agar colony formation assay for control and ANXA2-KD MGC-803 and AGS cells. **C**–**E** ANXA2-KD and control MGC-803 cells were inoculated subcutaneously in NOD-SCID mice, tumor images are shown (**C**), and tumor size was measured every 5 days (**D**). Twenty days later mice were sacrificed and tumor were collected and weighted (**E**). **F** Representative images and quantification data of scratch wound-healing assay for control and ANXA2-KD GC cells transfected with control or YES1 expression plasmid at 0 and 24 h after wound scratch. **G** Representative images and quantification data of the trans-well migration and invasion assay for control and ANXA2-KD GC cells transfected with control or YES1 expression plasmid. **H** Representative images and quantification data of the trans-well migration and invasion assay for AGS-YES1-KD cells transfected with control, WT and MT-ANXA2 expression plasmid respectively. **I** Western blot for the expression of indicated proteins in control and ANXA2-KD GC cells transfected with control, WT-ANXA2, or MT-ANXA2-Y24F expression plasmid for 48 h. **J** The representative images and quantification data of scratch wound-healing assay for control and ANXA2-KD GC cells transfected with control, WT-ANXA2 or MT-ANXA2-Y24F expression plasmid at 0 and 24 h after wound scratch. **K** The representative images and quantification data of the trans-well invasion assay control and ANXA2-KD GC cells transfected with control, WT-ANXA2, or MT-ANXA2-Y24F expression plasmid.

To define the role of ANXA2 in GC, we compared the cell proliferation rates, colony formation, migration and invasion among ANXA2-KD and control GC cells using MTT assay, soft agar colony formation assay, scratch wound-healing assay and trans-well invasion assay. We found that KD of ANXA2 in AGS and MGC-803 significantly decreased cell proliferation (Fig. 5A), colony formation (Fig. 5B), GC development in MGC-803 xenograft mouse models (Fig. 5C–E), migration, and invasion ability (Fig. 5F, G). These results suggest that ANXA2 promotes GC proliferation, growth, and invasion.

To examine whether ANXA2 mediates YES1-driven GC cell proliferation and invasion, ANXA2-KD and control AGS and MGC-803 cells were transfected with control or YES1 expression plasmid, and the cell proliferation rates, colony formation, migration, and invasion were detected. We found that KD of ANXA2 completely blocked YES1 OE-induced GC cell migration and invasion (Fig. 5F, G), while only partially inhibited YES1 OE-induced GC cell proliferation and colony formation (Fig. S5A, B). Furthermore, YES1-KD AGS cells were transfected with control or plasmid expressing WT-ANXA2 or MT-ANXA2-Y24F, and the colony formation in cell plate and soft agar, as well as migration and invasion were examined. We found that OE of WT-ANXA2 or MT-ANXA2-Y24F in YES1-KD cells could not restore GC cell migration and invasion (Fig. 5H), however could partially restore GC cell proliferation and colony formation (Fig. S6A). These results suggest that ANXA2 mediates YES1-induced GC cell invasion and migration but not YES1-induced GC cell proliferation although ANXA2 itself promotes GC cell proliferation.

### Phosphorylation of ANXA2-Tyr24 is essential for ANXA2-driven GC cell migration and invasion

To examine the role of ANXA2 (Tyr24) phosphorylation in GC cells, ANXA2-KD and control AGS and MGC-803 cells were transfected with WT-ANXA2 or MT-ANXA2-Y24F expression plasmid, and the restoration of ANXA2 expression in these cells was confirmed by WB (Fig. 5I). The cell proliferation rates, anchorage-independent growth, migration and invasion were analyzed. We found that MT-ANXA2-Y24F had similar potency to WT-ANXA2 to restore the proliferation and colony formation of ANXA2-KD cells (Fig. S7A, B), while only WT-ANXA2 rather than MT-ANXA2-Y24F efficiently restored the migration and invasion of ANXA2-KD cells (Fig. 5J, K). These data suggest the p-ANXA2-Y24 is essential for ANXA2-driven GC cell migration and invasion, but is dispensable for ANXA2-induced GC cell proliferation.

### YES1–ANXA2 pathway activation is manifested in human GC and related to GC recurrence

**Fig. 6:**
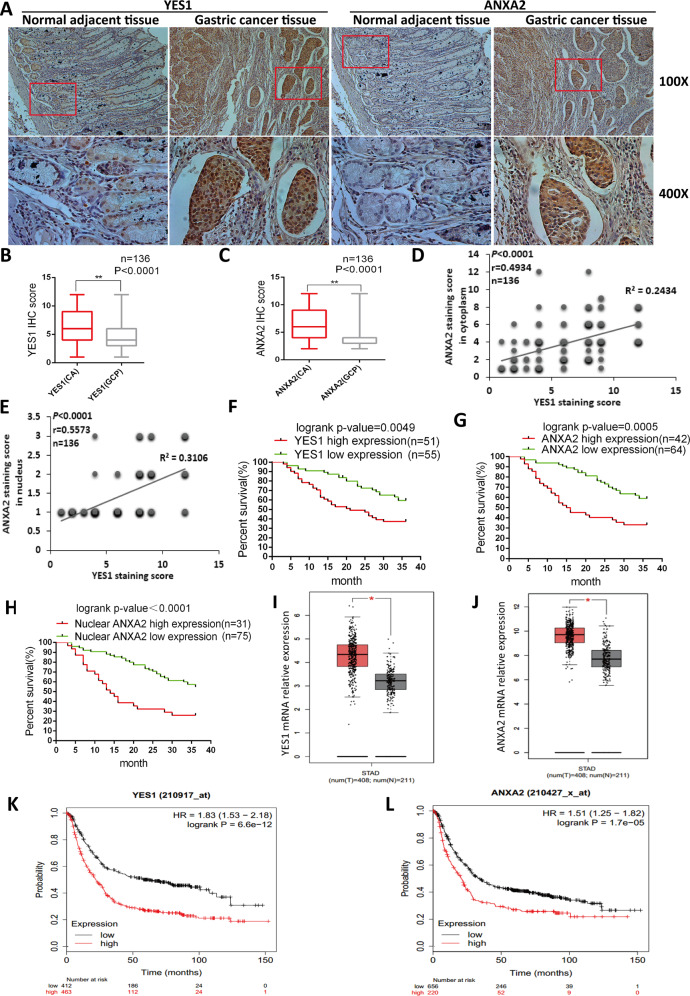
The clinical relevance of YES1–ANXA2 axis in human GC. **A** Representative images of the IHC staining for the expression of YES1 and ANXA2 in human GC tissues and paired adjacent normal gastric mucosa. **B** Comparison of YES1 protein expression in human GC tissues and paired adjacent normal gastric mucosa. **C** Comparison of ANXA2 protein expression in human GC tissues and paired adjacent normal gastric mucosa. **D** The correlations between YES1 and cytoplasmic ANXA2 expression in human GC tissues (The size of spots reflects the number of samples). **E** The correlations between YES1 expression and ANXA2 nuclear staining in human GC tissues (The size of spots reflects the number of samples). **F** The correlations of YES1 expression with patient survival analyzed by Kaplan–Meier Survival curve. **G** The correlations of total ANXA2 expression with patient survival analyzed by Kaplan–Meier Survival curve. **H** The correlations of nuclear ANXA2 expression with patient survival analyzed by Kaplan–Meier Survival curve. **I** Comparison of YES1 mRNA expression in GC tissues (*n* = 408) and normal tissues (*n* = 211) analyzed via the GEPIA (Gene Expression Profiling Interactive Analysis). Data are from GEPIA (http://gepia.cancer-pku.cn) database. **J** Comparison of ANXA2 mRNA expression in GC tissues (*n* = 408) and normal tissues (*n* = 211) analyzed via the GEPIA. Data are from GEPIA (http://gepia.cancer-pku.cn) database. **K** The correlations of YES1 expression with patient survival. Data are from the Kaplan–Meier Plotter (http://kmplot.com) databases (YES1 (210917_at)). **L** The correlations of ANXA2 expression with patient survival. Data are from the Kaplan–Meier Plotter (http://kmplot.com) databases (ANXA2 (210427_x_at)).

We analyzed 136 cases of human GC samples and paired adjacent normal gastric mucosa by IHC for the expression of EphA2, YES1, and ANXA2. We found that the expression of EphA2 was significantly related to tumor invasion and metastasis in our previous article [9]. The correlation between the expression of YES1 and ANXA2 with the clinical characteristics was analyzed and summarized in Supplementary Table 3 (Table S3). We found that the expression levels of YES1 and ANXA2 in the GC tissues were significantly higher than the adjacent normal gastric mucosa (Fig. 6A–C). The expression levels of cytoplasmic ANXA2 and nuclear ANXA2 were positively correlated with YES1 expression in GC tissues (Fig. 6D, E), as well as the total ANXA2 (Fig. S8A). We also analyzed the correlations between EphA2 and YES1 or ANXA2 expression, and we found that the expression of EphA2 was positively correlated with YES1 expression in GC tissues (Fig. S9A), while it was not significantly related to ANXA2 staining (Fig. S9B). The expressions of YES1 and ANXA2 were significantly related to tumor invasion, regional metastasis and GC relapse during 3-year follow-up period (Table S3). In addition, the expression of YES1, ANXA2, and nuclear ANXA2 was significantly correlated with the overall survival of GC patients during 3-year follow-up period (Fig. 6F–H). Consistently, the results from databases also indicated that the expression levels of YES1 and ANXA2 mRNA in the GC tissues were significantly higher than the adjacent normal gastric mucosa (Fig. 6I, J), and is related to the survival rates of GC patients (Fig. 6K, L). These results strongly suggest that YES1–ANXA2 pathway activation is manifested in human GC and plays important roles in GC progression and metastasis.

## Discussion

**Fig. 7:**
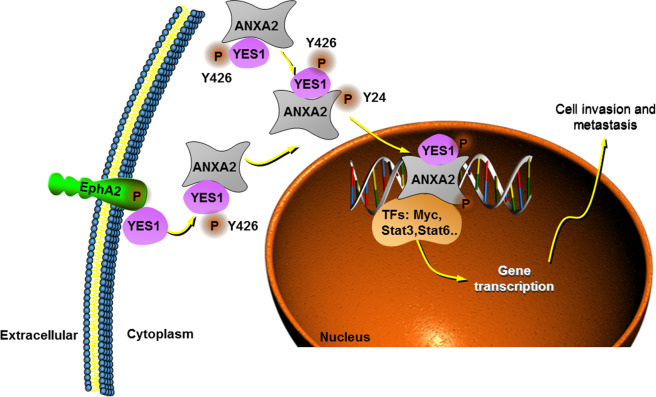
Schematic diagram of EphA2–YES1–ANXA2 pathway in GC growth and progression. EphA2 interacts with YES1 and phosphorylates YES1 at Tyr426 site that results in YES1 activation. Activated YES1 interacts with ANXA2 andphosphorylates ANXA2 at Tyr24 site that induces ANXA2 activation and increases ANXA2 nuclear distribution, leading to activation of the tumorpromoting transcription factors (such as Myc, Stat3, Stat6) that promotes GC invasion and metastasis.

GC is one of the most common malignancies worldwide with poor prognosis and high death rate [1, 2]. There are currently no effective therapies for unresectable advanced GC. GC local recurrence, gross peritoneal dissemination, direct invasion to other organs, and extensive distant organ metastases are the leading causes of death for GC patients [34]. Understanding the mechanisms that underlie the pathogenesis of GC progression and metastasis is needed to develop novel therapeutic approaches. In this work, we demonstrated EphA2–YES1–ANXA2 axis as a novel and important pathway that drives GC cell invasion and metastasis. In this chain of axis, EphA2 interacts with YES1 and phosphorylates YES1 at Tyr426 site that results in YES1 activation, activated YES1 interacts with ANXA2 and phosphorylates ANXA2 at Tyr24 site that induces ANXA2 activation and increases ANXA2 nuclear distribution, leading to activation of the tumor-promoting transcription factors [33, 35–38] that promotes GC invasion and metastasis (Fig. 7).

EphA2, as a key member of the RTKs, controls multiple physiological and pathological processes and is one of the promising targets for the treatment of cancers including GC [39]. In the early studies, we had demonstrated that EphA2 is overexpressed in GC and related to poor prognosis and chemotherapy resistance [9, 40, 41]. In present study, we show that YES1 is one of the major EphA2 binding proteins and phospho-substrates. EphA2 phosphorylates YES1 at Tyr426 site, leading to the activation of YES1 that promotes GC cell proliferation, migration, and invasion.

YES1 is a member of SRCs of nRTKs, which plays important roles in many cellular processes, such as cell growth, adhesion, survival, and differentiation [16, 17]. YES1 gene amplification occurs in some types of cancer and is a key mechanism of resistance to EGFR or HER2 inhibitors, while downregulation of YES1 inhibits cell growth in several malignancies, including colon carcinoma, rhabdomyosarcoma, and basal-like breast cancer [18]. In present study, we demonstrate that YES1 is highly expressed in human GC tissues and is positively correlated with tumor invasion, regional metastasis, relapse and the poor overall survival rate of GC patients. OE of YES1 increases while KD of YES1 decreases GC proliferation, growth, and metastasis in vitro and in vivo.

YES1 participates in many signaling pathways, in which YES1 activates different substrates, such as focal adhesion kinase, BCAR1, and paxillin [16, 17]. One of the well-known substrates of YES1 is YAP. YAP is a transcriptional coactivator in the Hippo pathway that plays important roles in tumor development and progression [9]. In this study, we show that YES1 interacts with ANXA2 and phosphorylates ANXA2 at Tyr24 site in a YAP1-independent manner, leading to ANXA2 activation and increased ANXA2 nuclear distribution. Consistently, the expression of ANXA2 and nuclear ANXA2 is positively correlated with YES1 expression in human GC tissues.

Annexin A2 is a member of the annexin family consisting of up to 160 unique annexin proteins, each of which consists of three domains: a divergent NH_2_-terminal, a C-terminal and a preserved domain making the core of the protein [22]. The three phosphorylation sites at the amino-terminal domain of Annexin A2 are crucial to its biological function and subcellular locations [23]. Ser11 phosphorylation of ANXA2 is associated with the binding of S100A10 [42]. Ser25 phosphorylation of ANXA2, which enriches in non-polysomal mRNP complexes in the perinuclear region and secretory vesicles, is involved in the process of mRNA banding and secretory process [43, 44]. p-ANXA2-Y24 is related to the localization of ANXA2 to the plasma membrane, endocytic membrane system and nucleus. ANXA2 has a nuclear export signal, however, no nuclear localization signal domain is contained [22, 45]. It has been reported that ANXA2 have been found to co-localize with different nuclear bodies, suggests that ANXA2 may enters the nucleus via interacting with and binding to nuclear speckles [46]. Nuclear ANXA2 may involve in DNA replication and DNA repair [47, 48], as well as regulate the transcription via interaction with some transcription factors, such as STAT6 and STAT3 [35, 37]. Our studies demonstrate that ANXA2 plays a critical role in promoting GC cell proliferation and metastasis, the expression of ANXA2 is positively correlated with tumor invasion, regional metastasis, relapse and the poor overall survival of GC patients. p-ANXA2-Y24 increases ANXA2 nuclear distribution in GC cells and is specifically related to GC cell migration and invasion.

As discussed above, each individual of EphA2, YES1, and ANXA2 plays important roles in GC cell proliferation, invasion, and metastasis. However, activation of EphA2–YES1–ANXA2 axis drives GC invasion and metastasis, while has minor effect on GC cell proliferation. YES1 is one of the major mediators for EphA2-induced GC cell proliferation, however, ANXA2 does not mediate YES1-induced GC cell proliferation although KD of ANXA2 suppresses GC cell proliferation, and therefore ANXA2 is not in the chain of the signaling cascade that mediates EphA2-to-YES1-induced GC cell proliferation. In our further investigation, it would be interesting to know the molecules that are downstream of YES1 and mediate the EphA2–YES1 axis-induced cell proliferation signaling in GC cells.

In conclusion, our present studies establish EphA2–YES1–ANXA2 axis as a novel pathway that drives GC invasion and metastasis, targeting this pathway would be an efficient way for the treatment of GC.

## Materials and methods

### Cell lines and antibodies and reagents

AGS and MGC-803 cells were purchased from Procell Life Science & Technology (Wuhan, China). GES-1, HGC-27, SGC-7901, BGC-823, MKN-45, and MKN-74 cell lines are kindly provided by Advanced Research Center and Cancer Research Institute of CSU. Authentication of all cell lines were confirmed by STR profiling. All cell lines have been examined to exclude the mycoplasma contamination. Antibodies and reagents were listed in Supplementary Materials. The primers used for the amplification of indicated genes by qRT-PCR were listed in Supplementary Table 4 (Table S4).

### Proximity ligation assay (PLA)

The PLA was employed to detect the indicated endogenous protein–protein interaction in situ (at distances <40 nm) in GC cells according to the kit instructions and as described previously [49].

### In vitro kinase assay

Recombinant human GST-YES1 (100 ng) was incubated with recombinant human active GST-EphA2 (100 ng), nonactivated BTN-EPHA2 (100 ng), or ANXA2 at 30 °C for 30 min in reaction buffer containing 20 mM HEPES (pH = 7.6), 20 mM MgCl2, 0.2 mM ATP, 2 mM DTT, 20 mM b-glycerophosphate, and 0.1 mM sodium orthovanadate as described previously [50]. After terminating the reaction by adding 30 μl of SDS-PAGE sample buffer, immunoblotting was performed.

### Statistical analysis

All in vitro experiments were performed in triplicate and at least three times. Data are presented as mean ± SD. Differences in data between two groups were analyzed with two-tailed student *t* test. The protein expression levels and clinicopathologic parameters were compared by chi-square test. Bivariate correlations between study variables were calculated by Spearman’s rank correlation coefficients. The correlations of indicated gene expression with patient survival were analyzed by Kaplan–Meier Survival curve. The log-rank test was applied to compare the prognostic significance of indicated gene on survival. Statistical analyses were performed with SPSS software program (version 21.0; IBM Corporation) and GraphPad Prism 6 (San Diego, CA). *P* < 0.05 was considered to be statistically significant (*P < 0.05; **P < 0.01).

The detailed materials, methods, and statistical analysis are described in the Supplementary Materials.

## Supplementary information

Supplement table 2

Supplement table 2-1

Supplementary material-R3
