# Correction to: EphA2–YES1–ANXA2 pathway promotes gastric cancer progression and metastasis

**DOI:** 10.1038/s41388-021-02163-z

**Published:** 2022-01-19

**Authors:** Linfeng Mao, Weijie Yuan, Kaimei Cai, Chen Lai, Changhao Huang, Yi Xu, Shangwei Zhong, Chen Yang, Ran Wang, Pengwei Zeng, Heyuan Huang, Zhikang Chen, Zihua Chen

**Affiliations:** 1 Department of General Surgery, Xiangya Hospital, Central South University (CSU), Hunan Changsha, China; 2 Department of Gastrointestinal Surgery, Xiangya Hospital, CSU, Hunan Changsha, China; 3 The Hunan Provincial Key Lab of Precision Diagnosis and Treatment for Gastrointestinal Tumor, Xiangya Hospital, CSU, Hunan Changsha, China; 4 Department of Colorectal and Anus Surgery, Xiangya Hospital, CSU, Hunan Changsha, China; 5 International Joint Research Center of Minimally Invasive Endoscopic Technology Equipment & Standardization, Xiangya Hospital, CSU, Hunan Changsha, China

**Keywords:** Gastric cancer, Metastasis

Correction to: *Oncogene* (2021) 40:3610–3623 10.1038/s41388-021-01786-6, published online 3 May 2021

After publication of this article, we noticed errors in Fig. 5G in the original manuscript, in which the images of the ANXA2-KD Vector (−) YES1(+) group were misused inadvertently. In this corrected version, we had replaced the incorrectly misused images with the correct original images and provided the correct Fig. 5 below. We confirm that the mistakes do not affect the results and conclusions of the study and apologize for any inconvenience caused by this mistake.

**Fig. 5:**
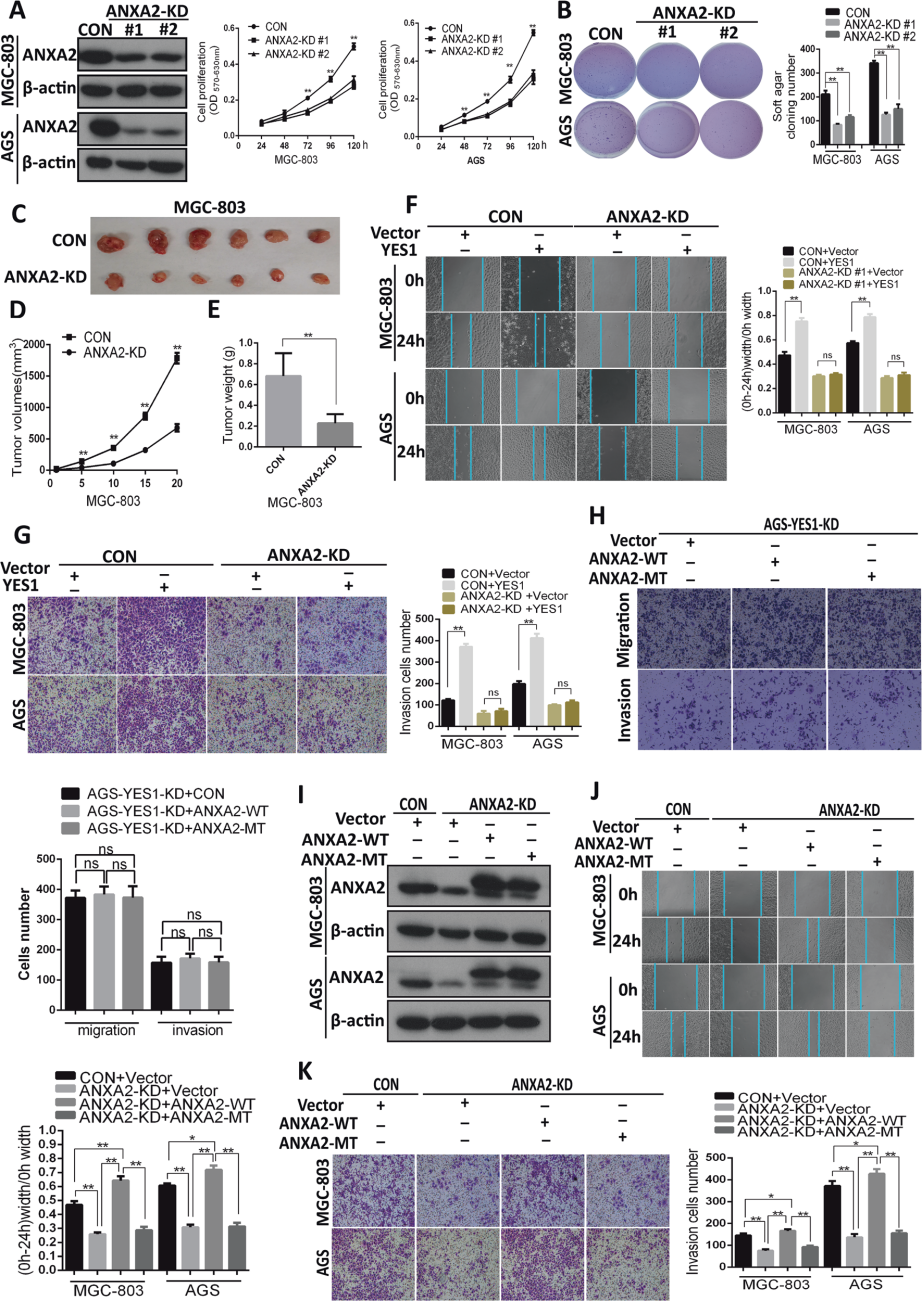
ANXA2-Tyr24 phosphorylation by YES1 promotes GC cell migration and invasion. **A** Western blot for the expression of indicated proteins in ANXA2-KD AGS and MGC-803 cells. MTT analysis for cell proliferation rates of control and ANXA2-KD MGC-803 and AGS cells. **B** Representative images of soft agar colony formation assay for control and ANXA2-KD MGC-803 and AGS cells. **C**–**E** ANXA2-KD and control MGC-803 cells were inoculated subcutaneously in NOD-SCID mice, tumor images are shown (**C**), and tumor size was measured every five days (**D**). Twenty days later mice were sacrificed and tumor were collected and weighted (**E**). **F** Representative images and quantification data of scratch woundhealing assay for control and ANXA2-KD GC cells transfected with control or YES1 expression plasmid at 0 and 24 h after wound scratch. **G** Representative images and quantification data of the trans-well migration and invasion assay for control and ANXA2-KD GC cells transfected with control or YES1 expression plasmid. **H** Representative images and quantification data of the trans-well migration and invasion assay for AGS-YES1-KD cells transfected with control, WT and MT-ANXA2 expression plasmid respectively. **I** Western blot for the expression of indicated proteins in control and ANXA2-KD GC cells transfected with control, WT-ANXA2, or MT-ANXA2-Y24F expression plasmid for 48 h. **J** The representative images and quantification data of scratch wound-healing assay for control and ANXA2-KD GC cells transfected with control, WT-ANXA2 or MT-ANXA2-Y24F expression plasmid at 0 and 24 h after wound scratch. **K** The representative images and quantification data of the trans-well invasion assay control and ANXA2-KD GC cells transfected with control, WT-ANXA2, or MT-ANXA2-Y24F expression plasmid.

The corrected Fig. 5 is given below:

The original article has been corrected.

