# Supplementary material for: EphA2–YES1–ANXA2 pathway promotes gastric cancer progression and metastasis
Source: Oncogene. 2021 May 3;40(20):3610–23. doi: 10.1038/s41388-021-01786-6 (PMC8134040; doi:10.1038/s41388-021-01786-6)
Supplement: Supplementary file 3 — Supplementary material-R3 [file 41388_2021_1786_MOESM3_ESM.docx]

**Supplementary Figures and Figure legends**


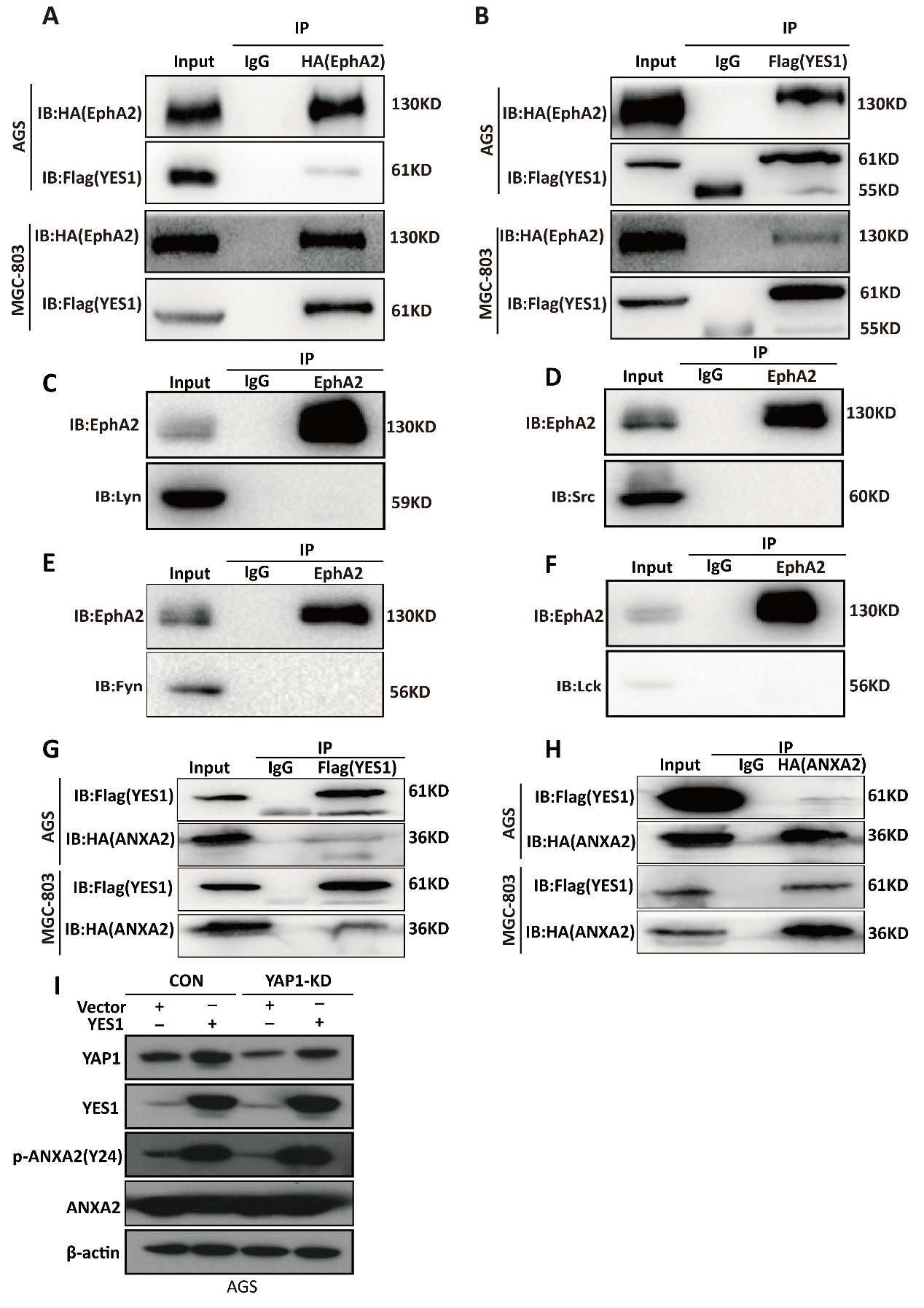


**Supplementary Figure 1 (A)** HA-EphA2 co-immunoprecipitation with Flag-YES1 in indicated GC cells. **(B)** Flag-YES1 co-immunoprecipitation with HA-EphA2 in indicated GC cells.


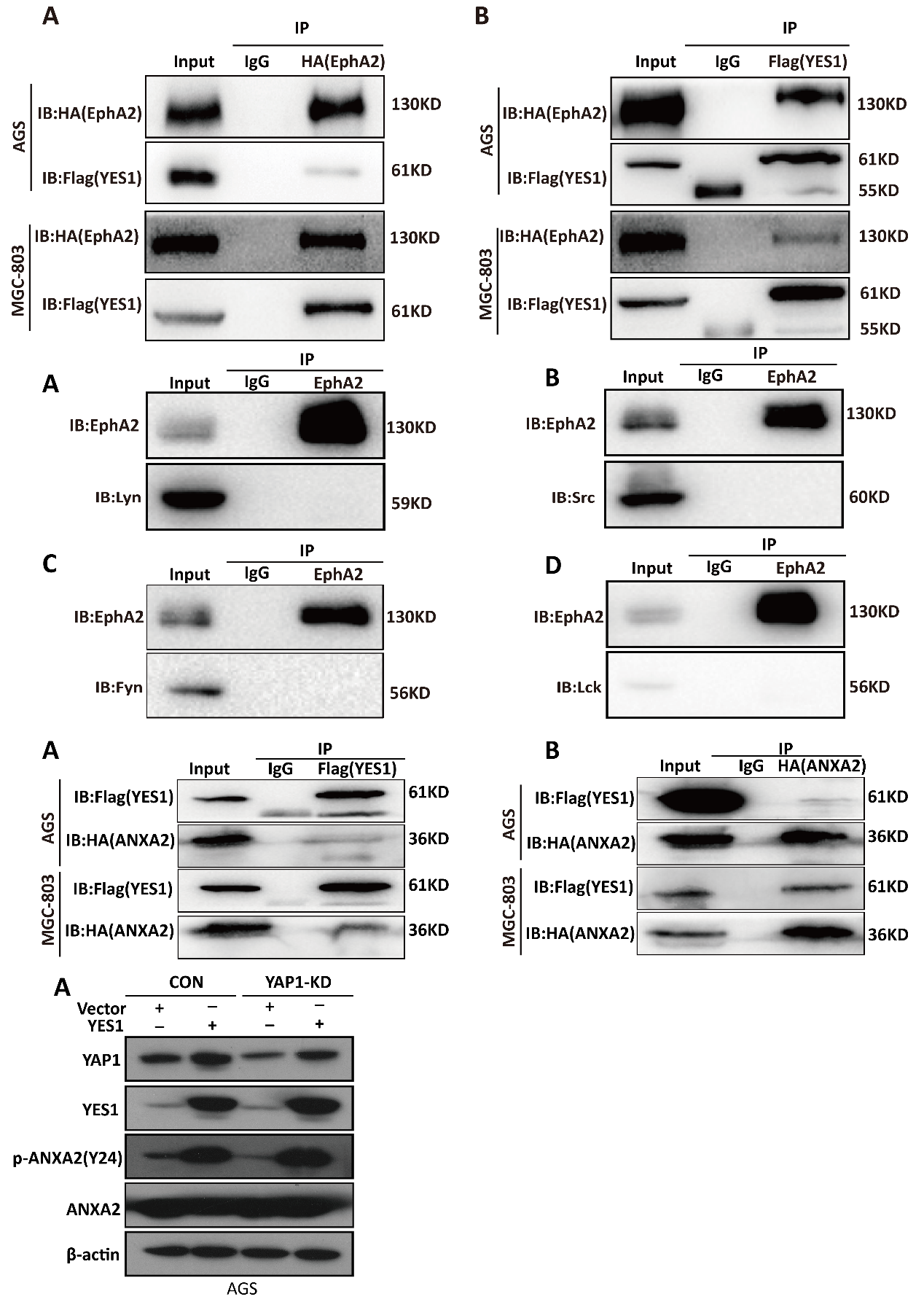


**Supplementary Figure 2** Co-immunoprecipitation for the interaction of EphA2 and other SFKs in AGS cells. **(A)** Co-immunoprecipitation for the interaction of EphA2 and Lyn in AGS cells. **(B)** Co-immunoprecipitation for the interaction of EphA2 and Src in AGS cells. **(C)** Co-immunoprecipitation for the interaction of EphA2 and Fyn in AGS cells. **(D)** Co-immunoprecipitation for the interaction of EphA2 and Lck in AGS cells.


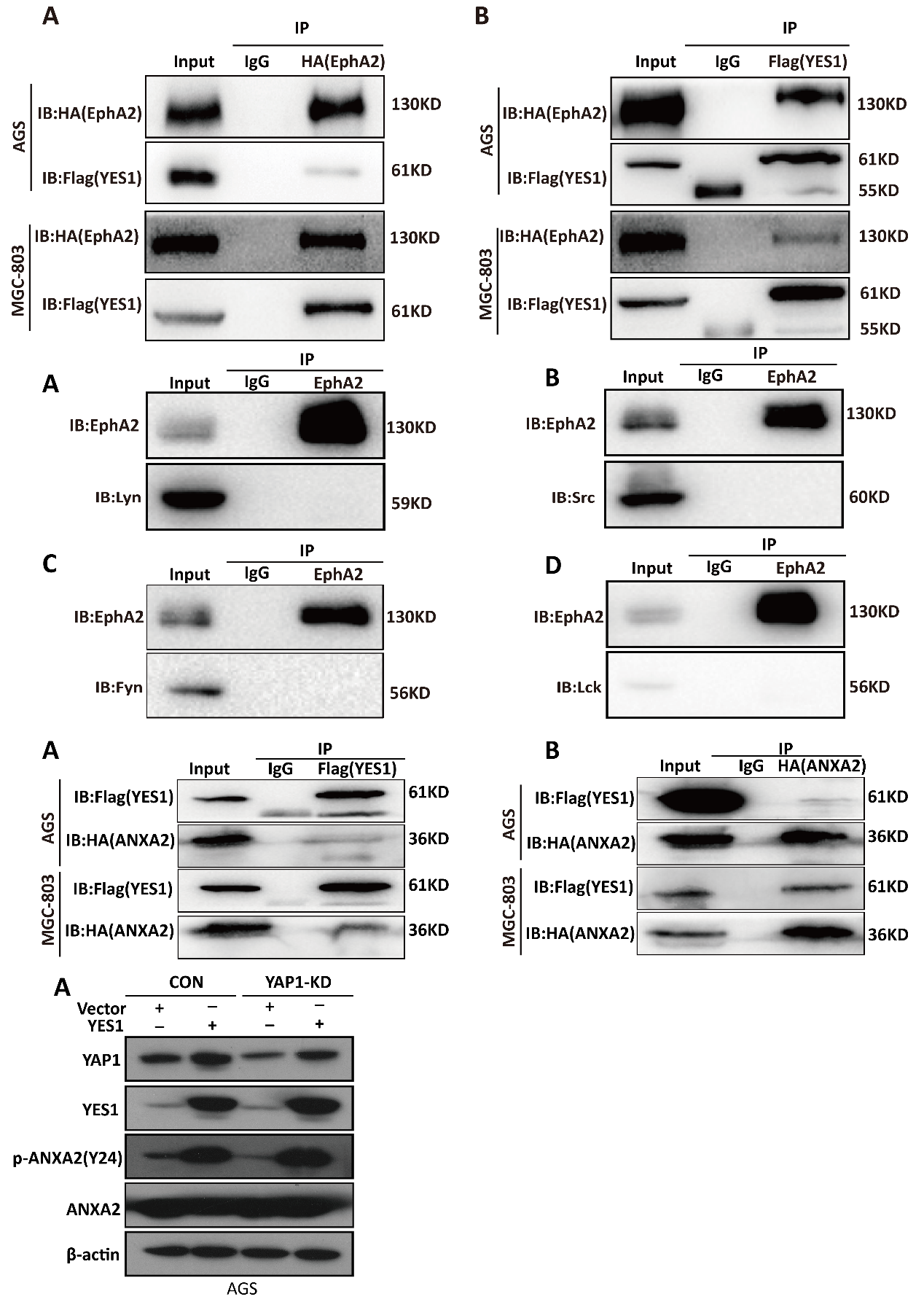


**Supplementary Figure 3 (A)** Flag-YES1 co-immunoprecipitation with HA-ANXA2 in indicated GC cells. **(B)** HA-ANXA2 co-immunoprecipitation with Flag-YES1 in indicated GC cells.


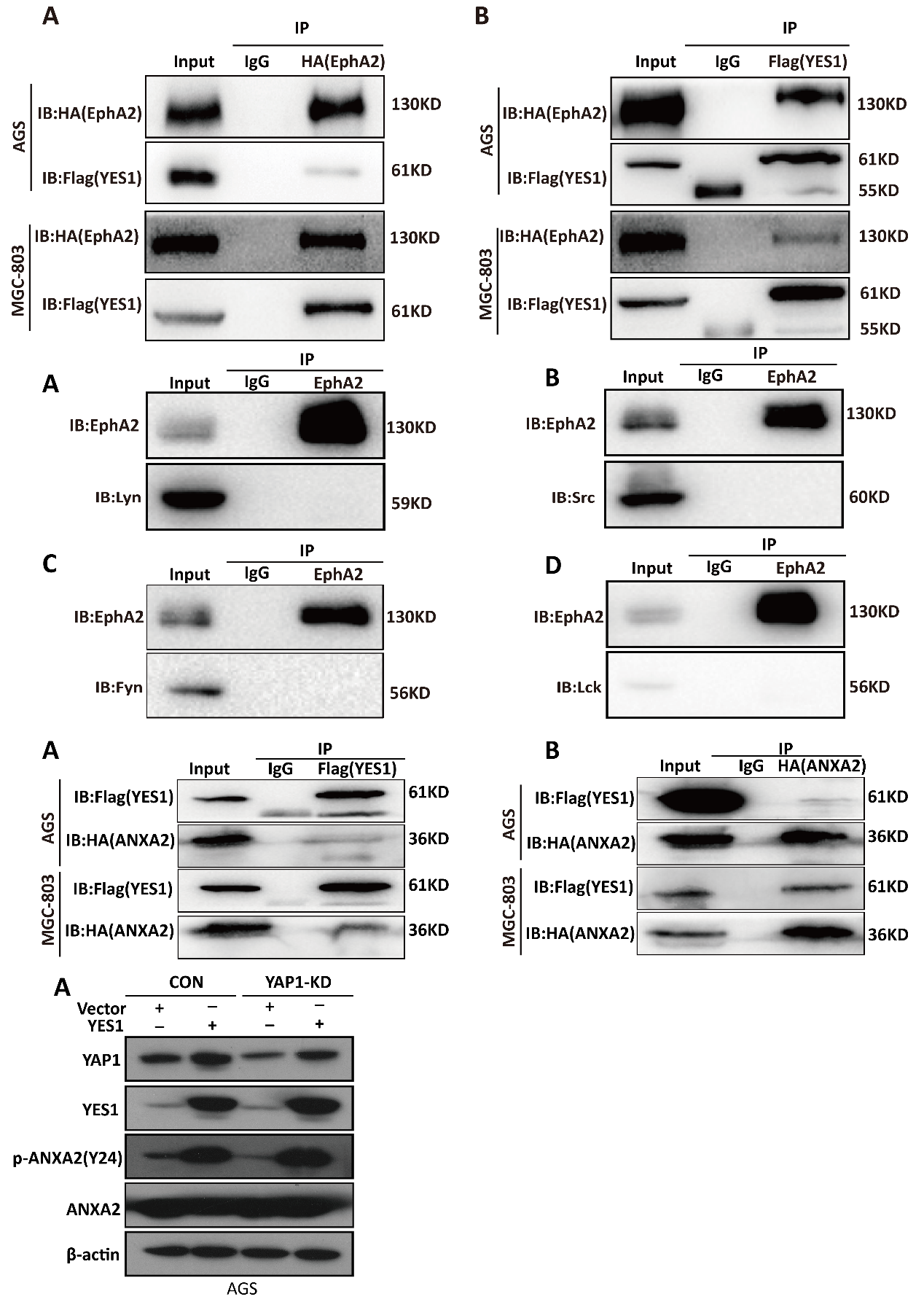


**Supplementary Figure 4 (A)** Western blot for the expression of indicated proteins in control and YES1-OE in YES1-KD or control AGS cells.


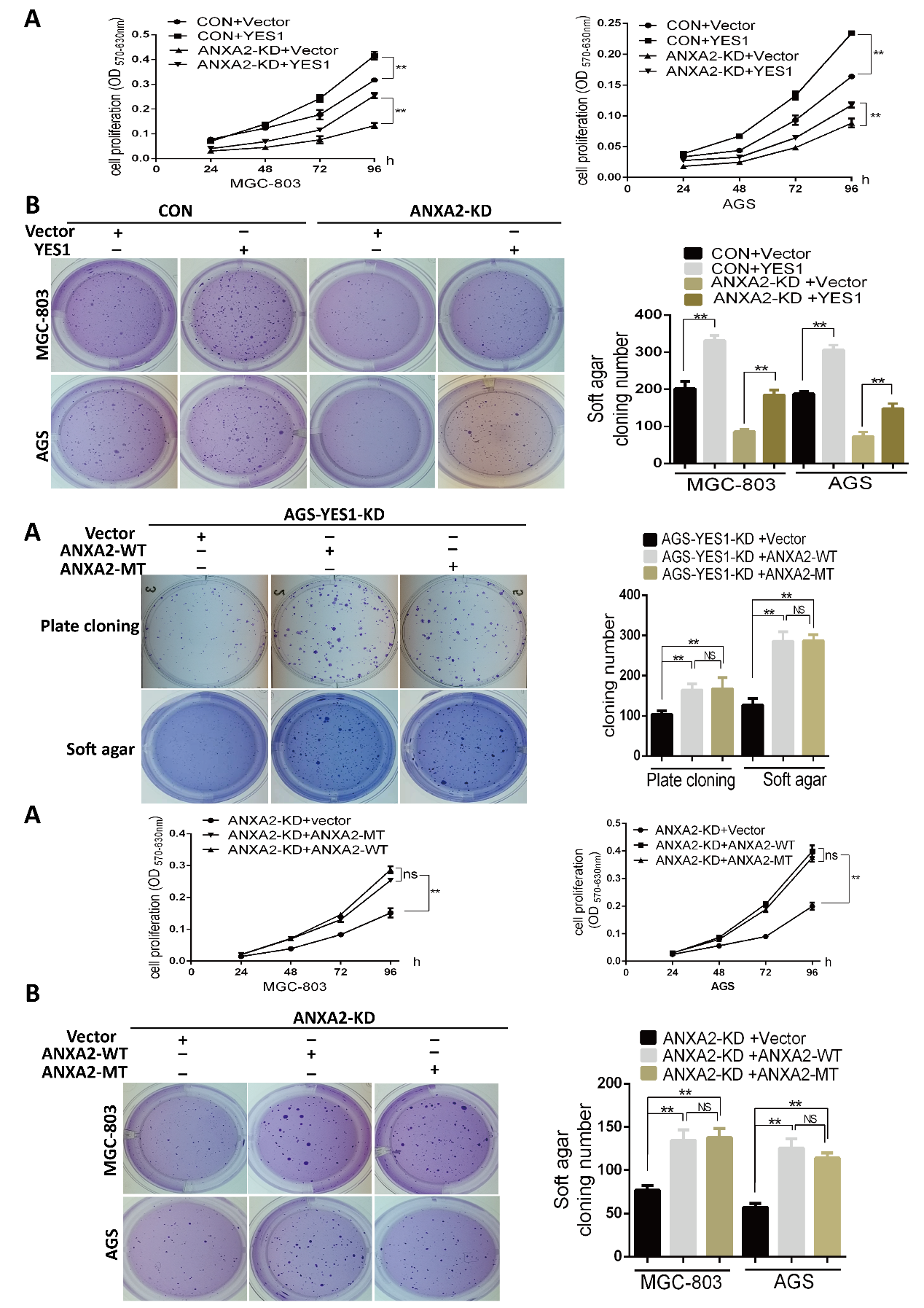


**Supplementary Figure 5 (A)** MTT analysis for cell proliferation rates of control and ANXA2-KD GC cells transfected with control or YES1 expression plasmid. **(B)** Representative images of soft agar colony formation assay and quantification data for control and ANXA2-KD GC cells transfected with control or YES1 expression plasmid.


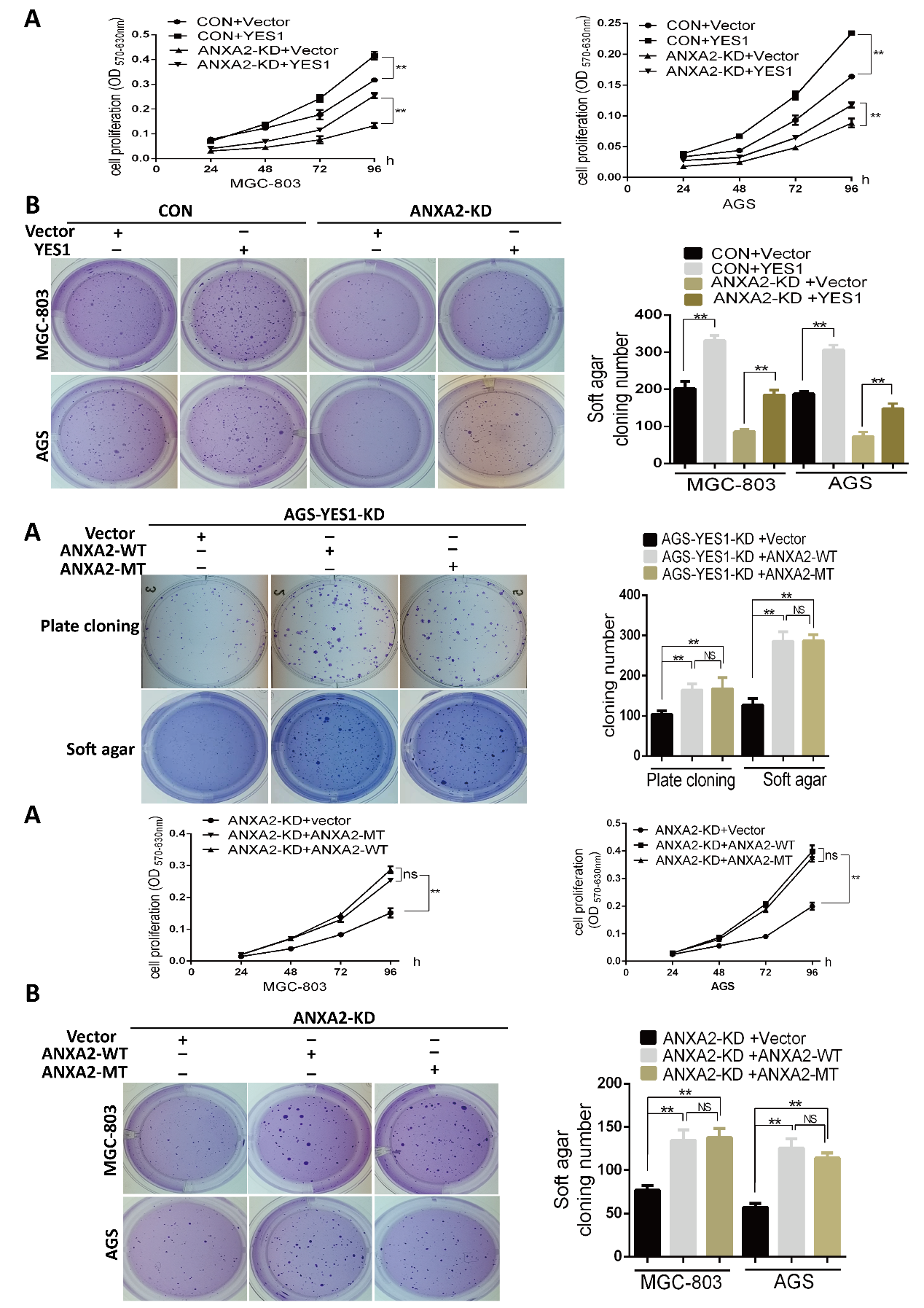


**Supplementary Figure 6 (A)** Representative images of plate colony formation assay, soft agar colony formation assay and quantification data for AGS-YES1-KD cells transiently transfected with control, WT ANXA2 and MT ANXA2 plasmids.


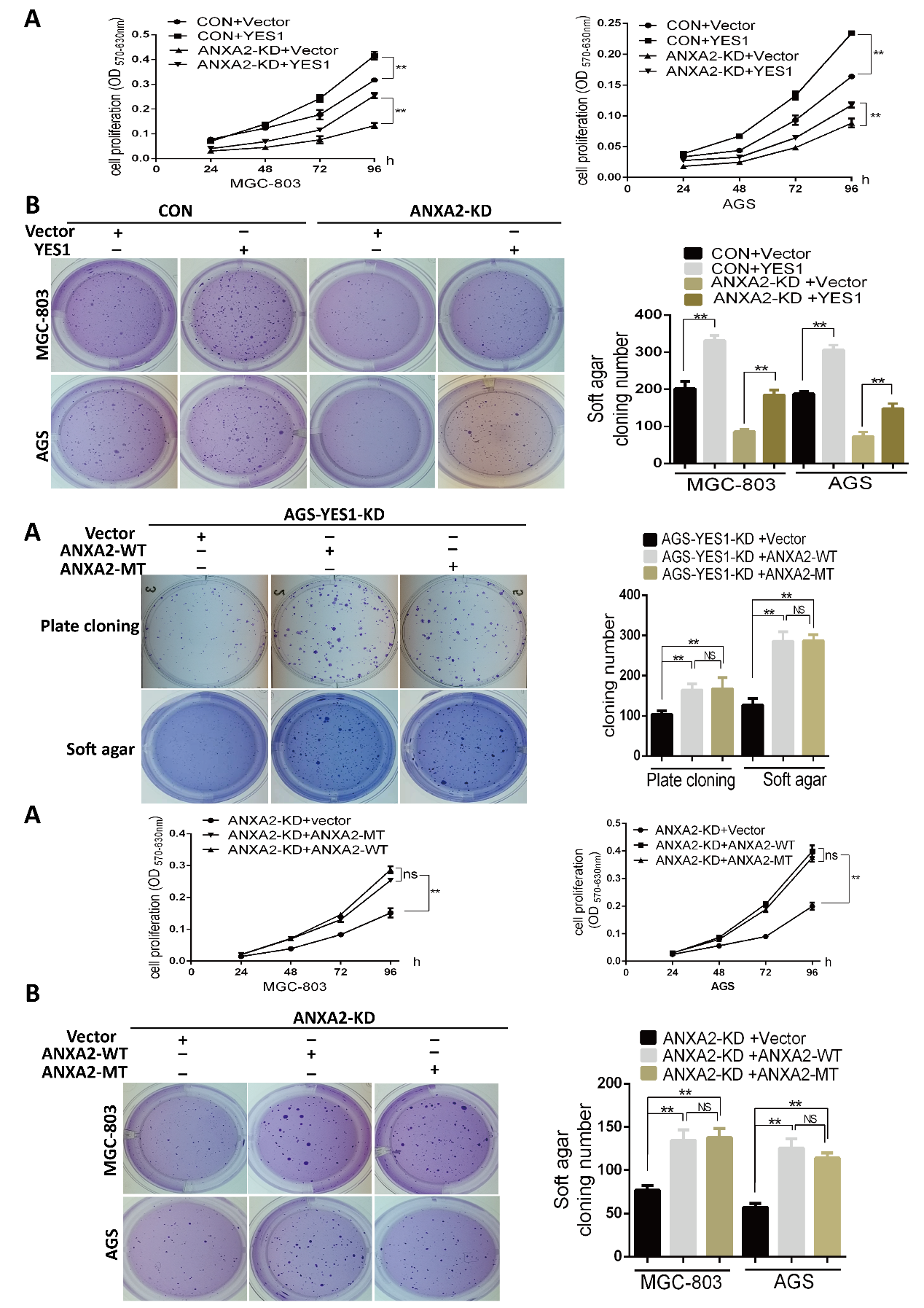


**Supplementary Figure 7 (A)** MTT analysis for cell proliferation rates of control and ANXA2-KD GC cells transfected with control, WT ANXA2 or MT ANXA2 expression plasmid. **(B)** Representative images of soft agar colony formation assay and quantification data for control and ANXA2-KD GC cells transfected with control, WT ANXA2 or MT ANXA2 expression plasmid.


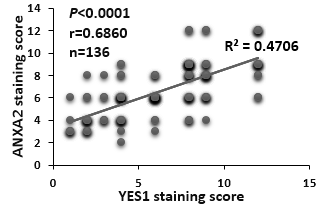


**Supplementary Figure 8 (A)** The correlations between YES1 and total ANXA2 expression in human gastric tumor tissues.


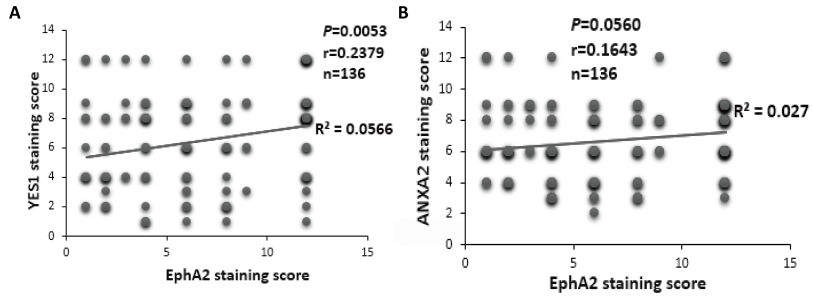


**Supplementary Figure 9 (A)** The correlations between EphA2 and YES1 expression in human gastric tumor tissues. **(B)**The correlations between EphA2 expression and ANXA2 staining in human gastric tumor tissues.

**Supplementary Tables**

**Supplementary Table 1. Proteins interact with EphA2 revealed in proteomic analysis**

| NO. | Swiss-Prot AC | Protein  name | MW (Da) | Coverage | Unique peptides | Scores |
| --- | --- | --- | --- | --- | --- | --- |
| 1 | Q13813 | SPTAN1 | 284.4 | 35.40 | 69 | 105.42 |
| 2 | P12814-2 | ACTN1 | 102.6 | 51.41 | 22 | 72.84 |
| 3 | O94832 | MYO1D | 116.1 | 47.12 | 40 | 66.29 |
| 4 | P29317 | EPHA2 | 108.2 | 32.79 | 27 | 64.09 |
| 5 | P06753-2 | TPM3 | 29.0 | 45.97 | 11 | 28.86 |
| 6 | Q5JP53 | TUBB | 47.7 | 45.07 | 2 | 21.93 |
| 7 | Q13885 | TUBB2A | 49.9 | 31.69 | 1 | 15.89 |
| 8 | Q05682-5 | CALD1 | 61.2 | 30.64 | 14 | 18.11 |
| 9 | Q14978-3 | NOLC1 | 73.7 | 16.86 | 10 | 15.71 |
| 10 | A0A0C4DGB6 | ALB | 69.2 | 10.60 | 6 | 13.91 |
| 11 | E9PCY7 | HNRNPH1 | 47.1 | 32.40 | 4 | 15.39 |
| 12 | Q13492-3 | PICALM | 66.4 | 20.33 | 10 | 18.10 |
| 13 | P17844-2 | DDX5 | 60.5 | 21.68 | 6 | 14.35 |
| 14 | P51114-2 | FXR1 | 60.8 | 21.52 | 8 | 9.86 |
| 15 | P56945-4 | BCAR1 | 77.7 | 17.73 | 8 | 11.45 |
| 16 | P07947 | YES1 | 60.8 | 9.21 | 4 | 3.53 |
| 17 | Q92841-1 | DDX17 | 72.3 | 15.38 | 4 | 12.60 |
| 18 | K7ESM5 | TUBB6 | 37.4 | 15.98 | 2 | 9.03 |
| 19 | O94973 | AP2A2 | 103.9 | 11.82 | 3 | 5.82 |
| 20 | F8VYE8 | PPP1CC | 34.9 | 31.25 | 1 | 1.84 |
| 21 | H0Y8G5 | HNRNPD | 29.6 | 26.15 | 4 | 6.89 |
| 22 | Q13045-2 | FLII | 138.4 | 8.07 | 7 | 0.00 |
| 23 | E9PQD7 | RPS2 | 25.2 | 34.04 | 7 | 8.21 |
| 24 | H3BLV0 | CD55 | 35.8 | 24.23 | 6 | 5.46 |
| 25 | P62140 | PPP1CB | 37.2 | 24.77 | 1 | 2.07 |
| 26 | M0QXS5 | HNRNPL | 58.4 | 17.17 | 7 | 6.51 |
| 27 | B7Z6D5 | DDX27 | 86.6 | 9.67 | 7 | 4.23 |
| 29 | P05141 | SLC25A5 | 32.8 | 19.13 | 6 | 7.66 |
| 29 | Q9NSY1 | BMP2K | 129.1 | 6.72 | 6 | 3.58 |
| 30 | Q8N1F7 | NUP93 | 93.4 | 7.33 | 6 | 0.00 |
| 32 | P02545-3 | LMNA | 70.6 | 11.99 | 6 | 5.61 |
| 33 | D6RG13 | RPS3A | 25.6 | 24.66 | 5 | 10.09 |
| 34 | A1L390-3 | PLEKHG3 | 128.2 | 7.14 | 6 | 6.60 |
| 35 | A0A1W2PQ43 | BCLAF1 | 80.6 | 15.65 | 8 | 4.72 |
| 36 | Q13435 | SF3B2 | 100.2 | 6.59 | 5 | 3.48 |
| 37 | P06748-2 | NPM1 | 29.4 | 23.40 | 5 | 7.21 |
| 38 | E9PNW4 | CD59 | 12.0 | 29.63 | 4 | 8.68 |
| 39 | B5MCT8 | RPS9 | 16.6 | 22.30 | 4 | 5.76 |
| 40 | Q7Z417 | NUFIP2 | 76.1 | 5.47 | 3 | 3.98 |
| 41 | O95171-3 | SCEL | 72.8 | 7.59 | 4 | 1.73 |

**Supplementary table 3 Correlation between the staining of YES1, ANXA2 and clinicopathologic characteristics in 136 cases of human gastric cancer tissues**

| 0parameter | | n | YES1 | | P | ANXA2 | | P |
| --- | --- | --- | --- | --- | --- | --- | --- | --- |
|  |  |  | - | + |  | - | + |  |
| Age(years) | |  |  |  |  |  |  |  |
| ≤60 | | 88 | 46 | 42 | 0.591 | 52 | 36 | 0.856 |
| ＞60 | | 48 | 22 | 26 |  | 27 | 21 |  |
| Gender | |  |  |  |  |  |  |  |
| male | | 99 | 45 | 54 | 0.123 | 57 | 42 | 1.000 |
| female | | 37 | 23 | 14 |  | 22 | 15 |  |
| Depth of tumor invasion |  | |  |  |  |  |  |  |
| T1-T2 | | 24 | 18 | 6 | 0.012 | 20 | 4 | 0.006 |
| T3-T4 | | 112 | 50 | 62 |  | 59 | 53 |  |
| Regional vascular metastasis | |  |  |  |  |  |  |  |
| Yes | | 51 | 14 | 37 | 0.000 | 19 | 32 | 0.000 |
| No | | 85 | 54 | 31 |  | 60 | 25 |  |
| Regional lymph node metastasis | |  |  |  |  |  |  |  |
| Yes | | 96 | 44 | 52 | 0.132 | 48 | 48 | 0.004 |
| No | | 40 | 24 | 16 |  | 31 | 9 |  |
| Regional nerve metastasis | |  |  |  |  |  |  |  |
| Yes | | 63 | 23 | 40 | 0.006 | 28 | 35 | 0.003 |
| No | | 73 | 45 | 28 |  | 51 | 22 |  |
| Histologic type | |  |  |  |  |  |  |  |
| Well and moderate | | 18 | 12 | 6 | 0.205 | 14 | 4 | 0.078 |
| Poor and undifferentiated | | 118 | 56 | 62 |  | 65 | 53 |  |
| Cancer Relapse (in 36 mouth) | |  |  |  |  |  |  |  |
| Yes | | 78 | 31 | 47 | 0.005 | 36 | 42 | 0.014 |
| No | | 28 | 20 | 8 |  | 21 | 7 |  |

*p* < 0.05 was considered to be statistically significant (chi-square test)

**Supplementary table 4. The primers used for the amplification of the two genes by qRT-PCR.**

| No. | Gene name | GenBank accession No. | Primer sequence |
| --- | --- | --- | --- |
| 1 | **YES1** | [**NM_005433**](http://www.ncbi.nlm.nih.gov/entrez/query.fcgi?cmd=Search&db=Nucleotide&term=NM_005433) | **F: GCCTGTCAGTACAAGTGTGAG**  **R: AAAGGCGTTACCCCTGAGGAT** |
| 2 | **GAPDH** | [**NM_001256799**](http://www.ncbi.nlm.nih.gov/entrez/query.fcgi?cmd=Search&db=Nucleotide&term=NM_001256799) | **F: GGAGCGAGATCCCTCCAAAAT**  **R: GGCTGTTGTCATACTTCTCATGG** |

**Supplementary Materials and Methods**

**Antibodies and reagents**

Anti-EphA2 antibody (#6997), Anti-[Yes1 (D9P3E) Rabbit mAb](https://www.cellsignal.cn/products/primary-antibodies/yes-d9p3e-rabbit-mab/65890?N=4294956287&Ntt=yes1&fromPage=plp) antibody(#65890), Anti-Src Family Antibody Sampler Kit( #9320), anti-HA tag (#3724), anti-Flag tag (#8146), anti-Phospho-Src Family (Tyr416) (#6943), Anti-ANXA2 antibody (#8235) were purchased from Cell Signaling Technology (Beverly, MA). Anti-GAPDH antibody(10494-1-AP), HRP-conjugated Affinipure Goat Anti-Mouse IgG(H+L) (SA00001-1), HRP-conjugated Affinipure Goat Anti-Mouse IgG(H+L) (SA00001-2) was from Proteintech Biotechnology. Anti-YES1 antibody (ab109365) was from Abcam. Anti-Ki-67 antibody (ZM-0166) was from Zhongshan Jinqiao Biotechnology (Beijing China). Anti-EphA2 antibody(sc-398832), Anti-Lamin A/C antibody (sc-376248), Anti-α-tubulin antibody(sc-8035), anti-β-actin antibody(sc-47778) and anti-p-Annexin II antibody (85. Tyr24) (sc-135753) were from Santa Cruz Biotechnology. The Duolink™ In Situ Red Starter Kit Mouse/Rabbit (Duo92101) for proximity ligation assay (PLA) was purchased from Sigma-Aldrich. Recombinant human active GST-EphA2 (Catalog No. 08-121), non-activated [BTN-EPHA2 (Catalog No. 08-421-23N)](https://www.carnabio.com/english/science/oneItem.cgi?id=143&pid=798) and active GST-YES1 (Catalog No. 08-175) protein were from Carna Biosciences (Kobe, Japan); recombinant human ANXA2 (Catalog No. 9409-AN-050) protein was from R&D Systems. The Mut Express MultiS Fast Mutagenesis Kit V2 (C215) was from Vazyme; The EphA2-HA (HG13926-CY), YES1-Flag (HG16953-CF) and ANXA2-HA (HG13517-CY) expression plasmids and the control plasmid were from Sino Biological. The YES1-Y426F, YES1-Y537F and ANXA2-Y24F mutant plasmid was generated by site-directed mutagenesis. YES1 and ANXA2 shRNA was constructed using a recombinant lentivirus gene delivery system by Sigma Aldrich as MISSION Lentiviral Transduction Particles (YES1 shRNA Clone ID: TRCN0000121062 and ANXA2 shRNA Clone ID: TRCN0000296322). Lipofectamine 3000 (L3000015) were purchased from ThermoFischer Scientific.

**Cell culture**

Human Gastric Mucosal Epithelial Cell Line (GES-1), Human GC cell line HGC-27, SGC-7901, BGC-823, MKN-45, and MKN-74 are kindly provided by Advanced Research Center and Cancer Research Institute of Central South University. Human GC cell lines AGS and MGC-803 were purchased from Procell Life Science & Technology (Wuhan, China). The cells were cultured in F-12 Kaighn’s Modification (for AGS), RPMI-1640 (for GES-1, SGC-7901, BGC-823,MGC-803, MKN-45 and MKN-74) and Dulbecco’s modified Eagle’s medium (DMEM, Gibco, Grand Island, NY, USA) (for HGC-27) supplemented with 10% fetal bovine serum (FBS, Biological Industries, Israel) and 1% Penicillin/streptomycin(complete medium) under a humidified atmosphere of 5% CO_2_ at 37°C. Authentication of all cell lines were confirmed by STR profiling. All cell lines have been examined to exclude the mycoplasma contamination.

**Transfection and transduction**

For transient transfection, GC cells were seeded into six-well plates or 6/10cm dish (for Co-immunoprecipitation). When the cells reached 80%-90% confluence, the plasmids were transfected into the cells with Lipofectamine 3000 according to its instructions. After 48 h, the cells were collected for different experiments. For establishing overexpression/knockdown stable cell lines, GC cells seeded in 24-well plates were transfected with plasmids or infected with shRNA lentiviral virus particles. 48 h later cells were reseeded into 10 cm cell culture dish and treated with selection antibiotics. The overexpression or knockdown efficiency of selected colonies was verified by western blot.

**Co-immunoprecipitation and Western blot**

Cells were lysed in ice-cold lysis buffer (50 mM Tris-HCl, pH 7.5, 250 mM/300 mM NaCl, 3 mM EGTA, 3 mM EDTA, 1% Triton X-100, 0.5% NP40, 10% glycerol, 2 mM DTT, 1 mM PMSF, 0.1 mM sodium vanadate, 2 mM PNPP, 1X Proteases inhibitors cocktail (A32963 ThermoFischer Scientific Rockford) for 30-60 min, sonicated every 5 minutes and centrifuged at 12,000 × g for 10 min at 4 °C. The supernatant was collected and protein concentration was measured by BCA protein assay kit (Thermo Scientific, Rockford, IL, USA).

For IP or co-IP, Aliquots containing 1 mg of protein cell lysates were incubated with specific antibodies overnight at 4 °C, 40 μl of protein A/G-plus agarose beads (Santa Cruz Biotechnology, Santa Cruz, CA, USA) were added into the lysates and incubated for more than 2h. Beads were then washed four times in lysis buffer, and then subjected to western blotting. Cytoplasmic and nuclear protein was extracted using Cytoplasmic and Nuclear Protein Extraction Kits (Thermo Scientific, Waltham, MA) according to its instructions. For western blotting, the same amount samples were run on SDS-PAGE gels and transferred onto Immobilon-P PVDF membranes (Merck Millipore, Billerica, MA, USA). After blocking with 5% skim milk in TBST for 1 h, the membranes were incubated with primary antibodies overnight at 4 °C and then the secondary antibody for 1h. Immunoreactive bands were detected using Enhanced Chemiluminescence (ECL) system (Bio-Rad, Hercules, CA, USA) or film exposure.

**Immunofluorescence**

After cells are transfected with plasmids for 48 h, they were plated onto the coverslips and incubated at 37 °C for 24 h, then fixed for 2 h in 95% ethanol, permeabilizated with 0.1% Triton X-100 for 20 min and processed for immunofluorescence. HA-EphA2 were stained with rabbit anti-HA (1:500), followed by Goat AntiRabbit IgG H&L (DyLight® 650) secondary antibodies, and Flag-YES1 was stained by mouse anti-Flag antibody, followed by Goat Antimouse IgG H&L (DyLight® 488) secondary antibodies. The primary antibodies ANXA2/p-ANXA2(Y24), which were diluted (ANXA2 1:200 rabbit), p-ANXA2(Y24) 1:100 mouse) in PBS with 5% bovine serum albumin, were applied overnight at 4°C. Goat Anti-Mouse IgG H&L (DyLight® 488) and Goat AntiRabbit IgG H&L (DyLight® 488) secondary antibodies (Abcam) were applied for 30 min. After washing thrice with PBS, DAPI was applied for 5 min at room temperature, and followed by washing thrice with PBS. The images were captured by Laser confocal imaging system or a fluorescence microscope.

***In vitro* kinase assay**

Recombinant human GST-YES1(100ng) was incubated with recombinant human active GST-EphA2(100ng), non-activated [BTN-EPHA2](https://www.carnabio.com/english/science/oneItem.cgi?id=143&pid=798)(100ng) or ANXA2 at 30℃ for 30 min in 30μl of reaction buffer containing 20mM HEPES(pH=7.6), 20mM MgCl2, 0.2mM ATP, 2mM DTT, 20mM b-glycerophosphate, and 0.1mM sodium orthovanadate. After terminating the reaction by adding 30μl of SDS–PAGE sample buffer, immunoblotting was performed.

**Quantitative Real-time PCR**.

qRT-PCR was employed to detect the relative mRNA expression of indicated genes in the indicated cells as described previously [10]. The primers are presented in the Supplementary Table 4. The relative levels of the target gene mRNAs were expressed as the ratio with GAPDH.

**Cell proliferation assay**

Cell proliferation was measured using a CCK-8 kit (C0038 beyotime Biotechnology Shanghai) or by MTT assay as described previously[9]. The indicated cells were seeded into 96-well plates at a density of 1.5/2 × 10^3^ cells per well in 100 μl complete medium. At different time points, medium was removed, 10 μl of CCK8 dye diluted in 100 μl RPMI-1640/DMEM was added. The spectrometric absorbance at the wavelengths of 450 was determined with a microplate reader (Tecan, Morrisville, NC). The assay was performed three times in triplicate.

**Plate/****Soft agar colony formation assay**

For plate colony formation assay: 400/well indicated cells were seeded into six-well plates and incubated with complete medium, replaced complete medium every 5 days. After 14 days, cell colonies were fixed with methanol and stained with 0.5% crystal violet (Sigma Aldrich), and the colony formation was determined by counting the number of stained colonies. For soft agar colony formation assay, which is used to evaluate cell anchorage-independent growth ability, briefly, prepared 2% noble agar by adding 2 g of noble agar to 100 ml of deionized water then Heated to dissolve it until agar is completely dissolved. Prepare 0.7% noble agar by mix 2% noble agar with complete medium at a ratio of 1: 1.85, then added 600 μl 0.7% noble agar to 24-well plates as the bottom layer. After the bottom layer of agar had solidified, harvested indicated cells , counted cells and calculated the number of cells needed per well, diluted equal number (1500/2000 per well) in 300 μl complete medium and mixed with 300 μl 0.7% noble agar as the upper layer of agar containing cells and then added it over the bottom layer. After upper layer of agar containing cells had solidified, added 400 μl of complete medium over the upper layer of agar to prevent desiccation. After cell colonies formed, cell colonies were stained with 0.5% crystal violet, and the colony formation was determined by counting the number of stained colonies.

**Wound healing assay.**

Indicated cells were seeded into six-well plates and grown to a confluent monolayer. Cell monolayers were wounded using a sterile 200 pipette tip, and washed twice using PBS to remove cell debris and incubated in medium with 1% FBS. Cell migration was monitored at 0 h and 24 h under an inverted microscope. Images were captured at different time point. The cell migration rate (%) = (wound distance at 0 h-wound distance at 24 h)/ wound distance at 0 h.

**Transwell assay.**

Indicated cells (5 × 10^4^/well) diluted in serum-free medium containing 1% bovine serum albumin were seeded into 8.0 μm pore size transwell chambers (Millipore), either coated with BD Matrigel Basement Membrane Matrix (BD Bioscience, for invasion assays) or uncoated (for migration assays). Medium containing 10% FBS wdas used as a chemoattractant in the lower chamber. After incubation at 37 °C for 24 h, cells on the upper surface of the membrane were removed using cotton swabs and cells on the lower surface of the membrane were fixed with 4% methanol and dyed with 0.1% crystal violet and then counted in five randomly selected microscopic fields under a light microscope.

**Animal experiment**For the cancer cell xenograft study, six-week-old male NOD-SCID mice were randomly grouped and injected subcutaneously 2 × 106 YES1 overexpression or control MGC-803 cells(n=4) and YES1 shRNA knockdown or control AGS cells(n=6) in 100 μl PBS mixed with Matrigel (1:1). Tumor volume was measured every 6 days as previously described [11] and make sure that tumor size does not exceed 20mm in any direction. After 30 days, the mice were sacrificed according to protocols that approved by the Institutional Animal Care and Use Committee of Central South University (Changsha, China). The tumor mass was weighed. Immunohistochemistry and WB was performed on the mice tumor tissue to confirm the expression of YES1. Immunohistochemistry staining of KI-67 was performed to examine cell proliferation rates. To detect the role of YES1 in tumor metastasis, five-week-old male nude mice were randomly grouped(n=5) and the indicated cells were injected into the spleen of nude mice. After 30 days, the mice were sacrificed, metastasis nodules in livers of mice were counted. The study was approved by the Research Ethics Committee of Xiangya Hospital, Central South University (committee’s reference No.201403051).

**Human GC tissue microarray preparation and Immunohistochemistry**

Patient selection and tissue microarray preparation was described previously[9], the study was approved by the Research Ethics Committee of Xiangya Hospital, Central South University(committee’s reference No.201403168). Based on voluntary principles, all patients were informed the purpose of this study, they could freely decide whether they want to participate in the investigation or not and gave their written informed consent before inclusion in this study. In the third year of follow-up survey, 106 out of 138 patients were followed up, the other 32 patients were lost to follow-up. Immunohistochemical staining for YES1 (1:200), ANXA2 (1:200) was performed on the tissue microarray slides. Detailed protocols and methods were described previously[9]. The staining results were evaluated by two independent pathologists (double-blinded).

**Statistical analysis**
All in vitro experiments were performed in triplicate and at least three times. Data are presented as mean ± SD. Differences in data between two groups were analyzed with two-tailed student t test. The protein expression levels and clinicopathologic parameters were compared by chi-square test. Bivariate correlations between study variables were calculated by Spearman’s rank correlation coefficients. The correlations of indicated gene expression with patient survival were analyzed by Kaplan-Meier Survival curve. The log-rank test was applied to compare the prognostic significance of indicated gene on survival. Statistical analyses were performed with SPSS software program (version 21.0; IBM Corporation) and GraphPad Prism 6(San Diego, CA). P<0.05 was considered to be statistically significant (* P<0.05, ** P<0.01).
